# Real-time single-molecule tethered particle motion analysis reveals mechanistic similarities and contrasts of Flp site-specific recombinase with Cre and λ Int

**DOI:** 10.1093/nar/gkt424

**Published:** 2013-05-21

**Authors:** Hsiu-Fang Fan, Chien-Hui Ma, Makkuni Jayaram

**Affiliations:** ^1^Department of Life Sciences and Institute of Genome Sciences, National Yang-Ming University, Taipei 112, Taiwan and ^2^Section of Molecular Genetics and Microbiology, University of Texas at Austin, Austin, TX 78712, USA

## Abstract

Flp, a tyrosine site-specific recombinase coded for by the selfish two micron plasmid of *Saccharomyces cerevisiae*, plays a central role in the maintenance of plasmid copy number. The Flp recombination system can be manipulated to bring about a variety of targeted DNA rearrangements in its native host and under non-native biological contexts. We have performed an exhaustive analysis of the Flp recombination pathway from start to finish by using single-molecule tethered particle motion (TPM). The recombination reaction is characterized by its early commitment and high efficiency, with only minor detraction from ‘non-productive’ and ‘wayward’ complexes. The recombination synapse is stabilized by strand cleavage, presumably by promoting the establishment of functional interfaces between adjacent Flp monomers. Formation of the Holliday junction intermediate poses a rate-limiting barrier to the overall reaction. Isomerization of the junction to the conformation favoring its resolution in the recombinant mode is not a slow step. Consistent with the completion of nearly every initiated reaction, the chemical steps of strand cleavage and exchange are not reversible during a recombination event. Our findings demonstrate similarities and differences between Flp and the mechanistically related recombinases λ Int and Cre. The commitment and directionality of Flp recombination revealed by TPM is consistent with the physiological role of Flp in amplifying plasmid DNA.

## INTRODUCTION

Site-specific recombinases of the tyrosine family (YRs) bring about a wide variety of biological consequences ranging from alternative developmental pathways for bacteriophages, equal segregation of phage and bacterial chromosomes and transposition of conjugative mobile elements to copy number control of plasmids found in budding yeasts (1–6). The chemistry of recombination is performed by four subunits of a YR within the context of a synapsed pair of target DNA sites. The reaction takes place by the following sequential steps: (i) association of two recombinase monomers with a target site, (ii) interactions between the bound monomers to form a stable recombinase dimer with concomitant DNA bending, (iii) synapsis of the recombinase bound target sites, (iv) cleavage and exchange of the first pair of strands to form a Holliday junction intermediate, (v) isomerization of the Holliday junction and (vi) and the resolution of the junction into recombinant products (7–9). However, within these common mechanistic and conformational themes, subtle variations occur among individual recombinases (10,11).

The present study focuses on the mechanism of recombination by the Flp recombinase coded for by the *Saccharomyces cerevisiae* plasmid two micron circle. The biological function of Flp is to trigger a replication-coupled DNA amplification reaction, which corrects any decrease in plasmid copy number resulting from rare missegregation events (2,4). Our findings permit mechanistic features of Flp to be compared with those of the related phage recombinases, λ Int and P1 Cre. Flp and Cre are capable of recombining their target sites, *FRT* and *Lox*P, respectively, without the help of accessory factors. However, Int requires the assistance of integration host factor (IHF) for integrative recombination between the phage and bacterial attachment sites, attP and attB, respectively. The phage excision reaction by Int, between attL and attR sites to restore attP and attB, requires not only IHF but also the phage-coded excisionase (Xis). Flp and Cre, and to a more limited extent Int, have been applied as effective tools for bringing about directed rearrangements in genomes of prokaryotes and eukaryotes (12–17).

The active sites of YRs harbor, in addition to the tyrosine nucleophile, a conserved pentad of catalytic residues comprising two arginines, a lysine, a histidine and either a histidine or a tryptophan (18). In Flp, the pentad corresponds to Arg-191, Lys-223, His-305, Arg-308 and Trp-330. Flp stands apart from other well-characterized YRs in assembling a shared active site by the donation of the cleavage nucleophile (Tyr-343) from one monomer to the pro-active site assembled by a neighboring monomer (19,20). As a result, strand cleavage occurs in ‘*trans*’. Int and Cre, by contrast, house their active sites within a monomer and execute strand cleavage in ‘*cis*’. Flp and Cre also differ in their binding affinities for the respective target sites as well as in their rate constants and cooperativities of DNA association (21). Furthermore, mathematical modeling and simulations suggest that synaptic structures formed by Cre are more stable than those formed by Flp. The topological complexity of recombination products formed from negatively supercoiled plasmid substrates is higher in the case of Flp reactions compared with Cre reactions. This difference has been attributed to the smaller bend angle introduced in the target DNA by two bound Cre monomers and the resulting propensity for synapsis to occur within the same plectonemic branch of a supercoiled circle (22).

The binding of YRs to target DNA sites, the interactions among DNA bound YR subunits, the organization of the synaptic complex, the relative geometry of the paired target sites and the chemical and conformational steps of recombination have been studied by a variety of approaches. They include electrophoretic mobility shift assays, surface plasmon resonance, electron microscopy, atomic force microscopy, analytical ultracentrifugation, fluorescence resonance energy transfer (FRET), X-ray crystallography and single molecule analysis (23–35). Cumulatively, these investigations have provided a generally unified picture of the two-step strand cleavage and exchange mechanism underlying tyrosine recombination.

Single molecule investigations are particularly useful in revealing conformational and kinetic features of recombination that are obscured or averaged out during standard ensemble studies. For example, single molecule tethered particle motion (TPM) analysis of λ Int mediated attL × attR recombination from start to finish has yielded new insights into the individual reaction steps (34). In the productive pathway, commitment to recombination occurs early, following the rapid association of Int, IHF and Xis with DNA and rapid synapsis of the protein bound recombination partners. Synaptic complexes, which are stabilized by strand cleavage, complete recombination with nearly 100% efficiency, aided by the irreversibility of multiple reaction steps. ‘Abortive’ and ‘wayward’ nucleoprotein complexes are also formed, but do not mature into functional synaptic assemblies. Thus, a conformational filter operates at an early stage in recombination.

In a recent TPM investigation of Cre-mediated site-specific recombination, Fan has observed, in addition to the normal pathway, the operation of side-pathways as well as the assembly of non-productive nucleoprotein complexes (35). Moreover, the relatively stable Holliday junction intermediate makes the strand cleavage/ligation steps of Cre recombination reversible. By a combination of tethered fluorophore motion (TFM) and FRET, the rate limiting step has been inferred to occur after the junction has undergone isomerization (36). Additionally, this method has identified two non-functional synaptic complexes, one of which is distinct in conformation from that seen in crystal structures. They are consistent with the occurrence of wayward complexes, and the operation of functional filters early in recombination suggested by the TPM characterization of λ Int and Cre reactions (34,35).

In light of the non-conformity of Flp with other YR recombinases in active site organization and the differences between the physico-chemical and topological attributes of Flp and Cre reactions, we have now applied TPM to analyze the entire course of Flp-mediated inversion and deletion reactions between *FRT* sites occurring in individual DNA molecules. The outcomes reveal important kinetic and thermodynamic differences between Flp and Cre, despite their overall mechanistic similarity. Furthermore, a catalytically inactive Flp mutant reveals the similarity of Flp to λ Int, and its difference from Cre, in requiring strand cleavage for stabilizing the synapse. The irreversibility of the chemical steps between the initiation and completion of an Flp recombination reaction observed in the present study is consistent with the physiological role of Flp in promoting a committed step in yeast plasmid amplification.

## MATERIALS AND METHODS

### Proteins and their purification

Expression and purification of Flpe or Flpe(Y343F) were carried out using previously published protocols (37,38). Flpe contains four amino acid changes from native Flp (39) and is more active than native Flp at 37°C. These changes are Pro2Ser, Leu33Ser, Tyr108Asn and Ser294Pro. The concentration of Flpe or Flpe(Y343F) used in individual assays was 200 nM, which is well above the reported K_d_, ∼1.1 nM (21). This concentration was found to be optimal for Flp-*FRT* association without the disadvantage of interactions with non-specific DNA.

### Plasmids and DNA substrates used for TPM assays

Plasmids used for the arabinose inducible expression of Flpe or Flpe(Y343F) in *Escherichia coli* have been previously described (40). The head-to-head (inverted) and head-to-tail (direct) *FRT* sites were constructed using a derivative of a previously described plasmid PL451 (41). An *FRT* site was introduced into this plasmid between the BsmBI and BssHII restriction enzyme sites in either inverted or direct orientation with respect to a neighboring *FRT* site located ∼600 bp away. The resulting plasmids provided the templates for generating by PCR the 1168 bp long TPM substrates with the desired *FRT-FRT* orientation. One of the two primers used for DNA amplification was labeled with digoxigenin and the other with biotin at their 5′ ends. Control DNA molecules, 1168 bp in size but lacking *FRT* sites, were prepared by PCR using pBR322 plasmid DNA (New England BioLabs) as the template. DNA molecules containing a single *FRT* site, 1168 or 549 bp in length, were obtained by PCR using PL451 as the template. The primer sequences used in the PCR reactions are listed in Table 1.

**Table 1.** Primer sequences and plasmid templates used to obtain the experimental DNA substrates by PCR amplification

DNA substrate	Template	Primer sequences
Direct *FRT* sites	PL451*(direct)*	5′-DigN –GCTCACTCATTAGGCACCC
1168 bp DNA		5′-Bio- CGAAATTCTACCGGGTAGG
Inverted *FRT* sites	PL451*(inverted)*	5′-DigN –GCTCACTCATTAGGCACCC
1168 bp DNA		5′-Bio- CGAAATTCTACCGGGTAGG
Single *FRT* site	PL451*(single)*	5′-DigN –GCTCACTCATTAGGCACCC
1168 bp DNA		5′-Bio- CGCAGCTGTGCTCGACGTT
1168 bp DNA	pBR322	5′-DigN –GGCTGGCTGGTTTATTGC
		5′-Bio- GCACAGATGCGTAAGGAG
Single *FRT* site	PL451*(direct)*	5′-DigN –GCTCACTCATTAGGCACCC
549 bp DNA		5′-Bio- CAGCCATCTGTTGTTTGCC

PL451 (41) is the parent plasmid, which was engineered to harbor a pair of *FRT* sites spaced ∼600 bp apart in either the direct or the inverted orientation.

All DNA substrates used in the TPM assays were prepared by PCR.

The primer pairs for the amplification reactions were labeled at the 5′ ends with digoxigenin (DigN) in one case and biotin (Bio) in the other. A DNA substrate lacking *FRT* was prepared from pBR322 DNA as the template for PCR.

### Single-molecule TPM measurement and data analysis

Preparation of reaction samples and chambers for TPM assays, image capture, contrast enhancement and data analyses were performed as detailed in earlier publications (35,42,43). The length resolution for 1168 bp DNA molecules using a 40-frame averaging window is ∼150 bp, *P* < 0.05 (35). All data for Brownian motion (BM) were smoothed using a five-point adjacent averaging algorithm. Only those molecules whose BM amplitudes within a 40-frame averaging window satisfied the length criterion (*P* < 0.05) were chosen as substrates for subsequent measurements. Molecules that stuck to the glass surface (with BM < 26 nm) for >5 data points, which correspond to 200 frames or ∼6.7 s, were excluded from the analyses. Molecules that exhibited distorted movement, whose BM amplitude ratios along the X and Y coordinates fell outside the range of 0.8–1.2 at any time, were also omitted.

The dwell time distribution histograms were fitted to either a single exponential or a bi-exponential decay algorithm using the following formulae:

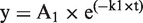


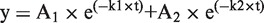



A_1_, A_2_, k1 and k2 are the fitting parameters determined by Origin 8.0.

### TPM assay conditions

All DNA molecules, labeled with digoxigenin and biotin at the 5′ ends on opposite strands, were attached to the surface of anti-digoxigenin coated coverslips. Then, the other end of such a DNA molecule was tethered to a streptavidin-coated 200 nm polystyrene bead, which served as the reporter of BM amplitude. The incubation was initiated by adding 30 µl of reaction buffer [50 mM Tris–HCl (pH = 7.5), 33 mM NaCl, 10 mM MgCl_2_, 5 mM dithiothreitol and 1 mg/ml bovine serum albumin (BSA)] containing 200 nM Flpe or Flpe(Y343F). Approximately 100 µl of reaction buffer containing sodium dodecyl sulfate (SDS), final concentration 0.05%, was flowed into the reaction chamber to quench the reaction. All experiments were performed at room temperature (22°C).

### Abbreviations and definitions

In the interest of clarity, the abbreviations repeatedly used in the text are explained in Table 2. The Flpe-associated DNA complexes analyzed by the TPM assays are described in Table 3.

**Table 2.** Abbreviations used in the text

Abbreviations	Expanded Description
BM	Brownian motion
TPM	Tethered particle motion
TFM	Tethered fluorophore motion
FRET	Fluorescence resonance energy transfer
YR	Tyrosine site-specific recombinase
Flp	Yeast plasmid coded tyrosine site-specific recombinase
Cre	Phage P1 coded tyrosine site-specific recombinase
λ Int	Phage λ coded tyrosine site-specific recombinase
*FRT*	Flp recombination target site
*LoxP*	Cre recombination target site
attL, attR	λ Int recombination target sites
attP, attB	Products of attL × attR recombination
IHF	Integration host factor
Xis	Excisionase protein

The abbreviations used in the manuscript, some of which may be unfamiliar to the non-expert, are explained.

**Table 3.** Definitions of Flpe-*FRT* complexes

Flpe-*FRT* complexes analyzed	Properties
Non-productive complex	Flp-bound *FRT* sites that do not form stable synapse; they have to dissociate before they can form the pre-synaptic complex.
Pre-synaptic complex	Flp-bound *FRT* sites that go on to synapse; they are the precursors of wayward as well as synaptic complexes.
Wayward complex	Flp-bound *FRT* sites that synapse but fail to proceed further in recombination; they may form synaptic complexes after first reverting to the pre-synaptic state.
Synaptic complexes	Flp-bound *FRT* sites that synapse and form the Holliday junction intermediate or complete recombination.

The different types of Flp recombinase bound DNA complexes characterized by the TPM analysis are described.

The non-productive and pre-synaptic complexes have the same BM amplitudes. The wayward complexes oscillate between the low amplitude of the synapsed state and the high amplitude of the pre-synaptic state before establishing stable synapse.

Once a stable synapse is formed, the wayward complexes can be distinguished from functional synaptic complexes by SDS challenge.

Although the Holliday junctions formed within the synaptic complexes retain their low BM state after protein dissociation, the wayward complexes return to the high BM state of the substrate molecules.

The linear recombinant product from an excision reaction by Flp has the same BM amplitude as the Holliday junction intermediate.

The product from an inversion reaction by Flp has the same BM amplitude as the parental molecule. Details are better explained in the appropriate sections of the text and under figures.

## RESULTS

### BM amplitudes report on changes in DNA length caused by recombinase binding or by strand exchange reactions

All of the assays reported in this study were performed using Flpe containing four amino acid substitutions in Flp (Pro2Ser, Leu33Ser, Tyr108Asn and Ser294Pro), which improve the thermal stability of the recombinase (39). The altered amino acids of Flpe are not expected to affect its mechanism, as revealed by the active site organization of Flp and Flpe in their respective crystal structures in association with DNA (20,44).

The rationale of the present analyses was based on the diagnostic BM amplitudes of a particle tethered to DNA as a function of the length of the DNA tether. In the TPM assays (34,35), changes in the BM amplitudes resulting from the addition of Flpe to a DNA substrate containing a pair of *FRT* sites were recorded and were correlated to changes in its length. These changes provided important insights into the recombination reaction, as they were characteristic of the binding of Flpe to *FRT* sites, synapsis of the Flpe bound sites, formation of the Holliday junction intermediate and the production of the mature recombination products.

### Changes in BM amplitudes in 1168 bp DNA molecules lacking *FRT* sites or containing one *FRT* site on Flpe addition

**Figure 1.**
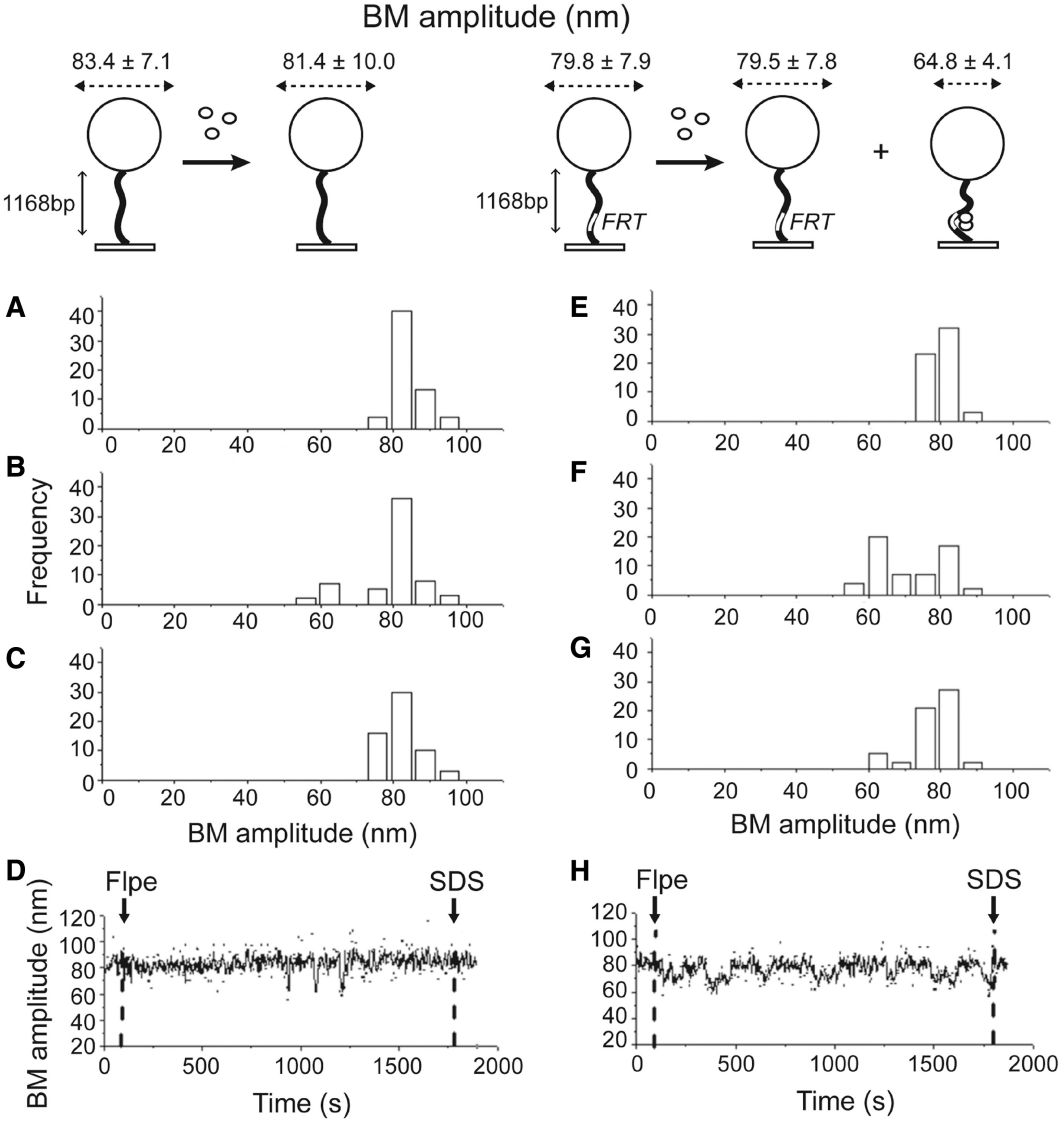
TPM analysis of the interaction between Flpe and DNA molecules lacking an *FRT* site or containing a single *FRT* site. The procedure for TPM measurements followed previously published protocols (see ‘Materials and Methods’ section) and is schematically diagrammed at the top. The black wavy line denotes a DNA molecule attached to a coverslip at one end and to a streptavidin-labeled polystyrene bead (200 nm in diameter; drawn as a sphere) at the other. The location of an *FRT* site on DNA is indicated by the small white rectangular box. Flpe monomers are shown as small white spheres. The dashed lines with arrowheads at either end represent the recorded BM amplitudes. Changes in these amplitudes are characteristic of the changes in DNA length as a result of Flpe binding, Flpe-mediated synapsis of *FRT* sites or strand exchange between the synapsed sites. (**A**) Control experiment with DNA lacking an *FRT* site in the absence of Flpe (BM amplitude = 83.4 ± 7.1 nm). (**B**) Response of the same DNA as in (A) to the addition of 200 nM Flpe. The histogram, fitted to a two-Gaussian distribution, gave BM amplitude peaks at 81.4 ± 10.0 nm (naked DNA) and 63.2 ± 8.0 nm (Flpe-bound DNA). The lower BM species was rare and short lived. (**C**) The BM amplitude distribution at 30 min after the addition of Flpe to DNA lacking *FRT*. For A–C, *N* = 61. (**D**) The BM amplitude trace of a single DNA molecule over a time course. The dashed vertical lines at the left and right indicate the addition of Flpe and SDS, respectively. (**E**) Control experiment with DNA containing a single *FRT* site without Flpe addition (BM amplitude = 79.8 ± 7.9 nm). (**F**) The same DNA as in (E) in response to the addition of 200 nM Flpe. Fitting the histogram to a two-Gaussian distribution gave BM amplitude peaks centered at 79.5 ± 7.8 nm and 64.8 ± 4.1 nm for naked DNA and Flpe-bound DNA, respectively. (**G**) BM amplitude distribution from DNA molecules containing a single *FRT* site after 30-min incubation with 200 nM Flpe. For E–G, *N* = 57. (**H**) The time course BM amplitude trace for a single DNA molecule from the time of Flpe addition (dashed vertical line at the left) to SDS challenge (dashed vertical line at the right). N refers (in subsequent figures as well) to the number of DNA molecules observed in each assay. In this analysis, as well as the ones to follow, statistical significance of the estimated BM amplitudes was given by *P* < 0.05.

First, we estimated BM amplitudes in 1168 bp long control DNA molecules containing no *FRT* sites with and without the addition of Flpe. In the absence of Flpe, the mean BM amplitude was estimated as 83.4 ± 7.1 nm (Figure 1A). On addition of 200 nM Flpe at room temperature, 14.75% of the molecules responded with a transient decrease in the BM amplitude that persisted for <20 s, contributing a small peak in the BM histogram (63.2 ± 8.0 nm, Figure 1B). However, measurements at 30 min after Flpe addition (81.4 ± 10.0 nm, Figure 1C) or a typical time course trace (Figure 1D) showed no stable change in BM amplitude caused by Flpe, indicating negligible non-specific interaction between Flpe and DNA molecules. Similar assays with 1168 bp DNA molecules containing a single *FRT* site gave a BM amplitude of 79.8 ± 7.9 nm (Figure 1E) without Flpe, which decreased to 64.8 ± 4.1 nm (Figure 1F) in 54.4% of the molecules as a result of binding of Flpe to the *FRT* site. Approximately 12.2% of the molecules remained in the low BM amplitude state at 30 min after Flpe addition (Figure 1G). Their behavior is illustrated by the time trace for such a molecule over this interval (Figure 1H). The shortening of the DNA by interaction with Flpe was consistent with a bend introduced in the *FRT* site as a result of Flp binding (45).

### Flp-mediated excision recombination between two *FRT* sites in head-to-tail (direct) orientation

**Figure 2.**
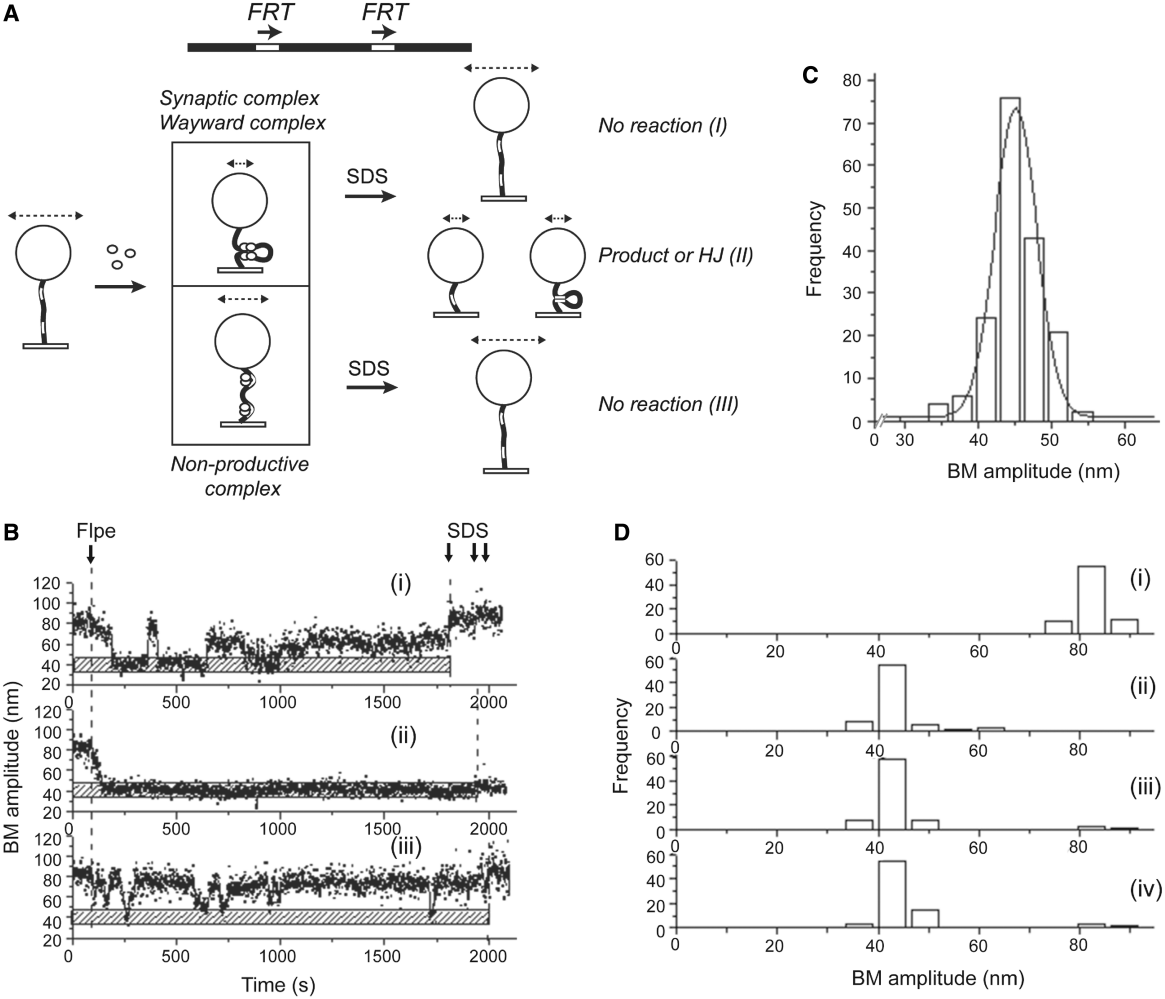
Analysis of pre-chemical and chemical steps following the association of Flpe with *FRT* sites in direct orientation*.* (**A**) The location of the *FRT* sites and their relative orientation in the DNA substrate are schematically indicated. The events following the addition of Flpe to DNA containing direct *FRT* sites (BM amplitude = 82.3 ± 7.4 nm) are outlined. Association of Flpe with the *FRT* sites may lead to non-productive complexes (BM amplitude = 59.2 ± 4.8 nm) or to synapsis of the Flpe-bound sites (BM amplitude = 42.0 ± 6.1 nm). SDS challenge distinguishes the wayward complexes (I) within the synapsed species from those that formed Holliday junctions and recombinant products (II). After SDS treatment, the non-productive (III) and wayward (I) complexes have the same BM amplitudes. The lengths of the double headed arrows above the beads schematically denote the BM amplitude differences of the various DNA species resulting from Flpe-*FRT* association and from strand exchange by Flpe. (**B**) The time traces exemplify (i) a molecule that synapsed after initial fluctuations but did not proceed further (a wayward complex), (ii) a molecule that not only synapsed but executed strand cleavage and ligation to form the Holliday junction or to complete recombination (a synaptic complex) and (iii) a molecule that failed to form a stable synapse (a non-productive complex). The stippled horizontal bar marks the expected mean BM amplitude for the synapsed complex, the Holliday junction or the linear recombinant product. (**C**) A histogram of the BM amplitudes (mean = 45.1 ± 5.7 nm) obtained from 549 bp DNA molecules representing the linear recombinant product (*N* = 177). (**D**) BM amplitude distributions before the addition of Flpe (mean = 82.3 ± 7.4 nm) (i); in response to the addition of Flpe and formation of non-productive (59.2 ± 4.8 nm) and synapsed (42.0 ± 6.1 nm) complexes (ii); after 30-min incubation with Flpe (iii); and following SDS challenge at the end of 30-min incubation (iv) (*N* = 75).

To characterize excision recombination by Flpe, we performed a similar analysis in 1168 bp DNA molecules containing two *FRT* sites in direct orientation and separated by 619 bp between their cross-over points (Figure 2A). With this orientation of sites, the final products are a deletion circle plus a shortened linear molecule attached to the bead.

The changes in BM amplitudes of DNA molecules as an immediate response to Flpe addition could be categorized into either a pre-synaptic state (that matured into a synapsed state) or a non-productive state (that failed to form stable synapse) as depicted in Figure 2A. The synapsed molecules could fluctuate between the low and high BM amplitudes (indicating wayward complexes), before establishing a stable low amplitude state (indicating more long-lived synapsis). Disruption of the non-covalent protein–protein and protein–DNA interactions by SDS challenge revealed the fate of the stably synapsed molecules during a 30-min reaction time. Molecules that failed to undergo recombination, wayward complexes, returned to the high BM amplitude matching that before Flpe addition (Figure 2A-I). Those molecules that formed the Holliday intermediate or completed recombination (excision of a 619 bp circle), representing the synaptic complex, retained the low BM amplitude (Figure 2A-II). Persistent non-productive Flpe-DNA complexes maintained their nearly steady BM amplitude, greater than that of synapsed molecules, before SDS treatment; they returned to the high BM amplitude of Flpe-free DNA molecules following SDS challenge (Figure 2A-III).

Single molecule time trace data generated from the analysis were characteristic of wayward complexes (Figure 2B-i), stable synaptic complexes that underwent strand cleavage and ligation (Figure 2B-ii) and non-productive complexes (Figure 2B-iii). The BM amplitude for 549 bp Viceroy DNA molecules (close mimics of the linear excision product constructed without recourse to Flpe recombination) was obtained as 45.1 ± 5.7 nm (Figure 2C). [The Viceroy is a mimic of the Monarch butterfly, and the Viceroy molecule in our context is a mimic of the linear excision product. The late Nick Cozzarelli referred to mimics of ‘butterfly’ replication intermediates as Viceroy molecules in Peter *et al.* (1998), Cell **94**, 819–827.] This value was set as diagnostic of any one of the following events: synapsis of the *FRT* sites, progression of the reaction to the Holliday junction intermediate or formation of excision recombinants. As diagrammed in Figure 2A, in all three cases, the reduction in the effective length of the DNA tethered to the bead would be the same. In the case of the inversion reaction, which is discussed separately, this BM amplitude represented either synapsis or Holliday junction formation, but not completion of recombination. This is because the inversion product was not altered in length from the starting substrate. Of 115 parental DNA molecules (1168 bp) with BM amplitudes of 75–90 nm (Figure 2D-i) obtained by averaging a 40-frame window, 75 responded to Flpe addition by displaying significantly lowered BM amplitudes (Figure 2D-ii). Five of these gave an average BM amplitude of 59.2 ± 4.8 nm (Figure 2D-ii), sufficiently close to 64.8 ± 4.1 nm observed for the control DNA molecules containing a single *FRT* site (Figure 1F). They were likely bound by Flpe at one or both *FRT* sites to form the non-productive complexes but did not proceed further into synapsis. The other 70 showed a decrease in BM amplitude to 42.0 ± 6.1 nm (Figure 2D-ii), consistent with synapsis, Holliday junction formation or successful recombination. Some molecules that formed the non-productive complexes (and failed at synapsis) returned to the high BM status of the molecules before Flpe addition, whereas those that formed the synaptic complexes maintained this state after 30-min incubation with Flpe (Figure 2D-iii). SDS-challenge at 30 min revealed that 93.3% of the synapsed molecules had either undergone Holliday junction formation or recombination (Figure 2D-iv). The remainder signified wayward complexes that were unsuccessful in advancing from synapsis to the Holliday junction stage. In a similar experiment, 91.4% of the synapsed complexes went on to form the Holliday junction or complete recombination within 10 min (Supplementary Figure S1A).

The dwell time histograms for molecules that responded to Flpe were fitted to a single exponential model to obtain the relevant kinetic parameters. The formation of non-productive complexes and pre-synaptic complexes occurred with association rate constants of (1.8 ± 0.1) × 10^5 ^M^−^^1 ^s^−^^1^ and (6.0 ± 0.3) × 10^4 ^M^−^^1 ^s^−^^1^, respectively (Supplementary Figure S1B-i and ii). Similarly, the rate constant representing the stability of the non-productive complex (Supplementary Figure S1C-i) was obtained as (4.0 ± 0.3) × 10^−^^2 ^s^−^^1^ (Supplementary Figure S1D-i). From the pooled dwell times of molecules in the pre-synaptic state, association rate constants of (1.7 ± 0.2) × 10^−^^2 ^s^−^^1^ and (4.9 ± 0.4) × 10^−^^2 ^s^−^^1^ for the wayward complexes (Type a; Supplementary Figure S1C-ii and D-ii) and the synaptic complexes (Type b; Supplementary Figure S1C-ii and D-iii), respectively, were obtained.

The TPM results demonstrate that synapsed molecules either form Holliday junctions or complete recombination with close to 100% efficiency. Based on the TPM characterization of *FRT* sites in the opposite orientation (described later in the text), we suspect that the recombinant product far exceeds the Holliday junction intermediate within the population of molecules in the low BM state.

### Flp-mediated inversion recombination between two *FRT* sites in head-to-head (inverted) orientation

**Figure 3.**
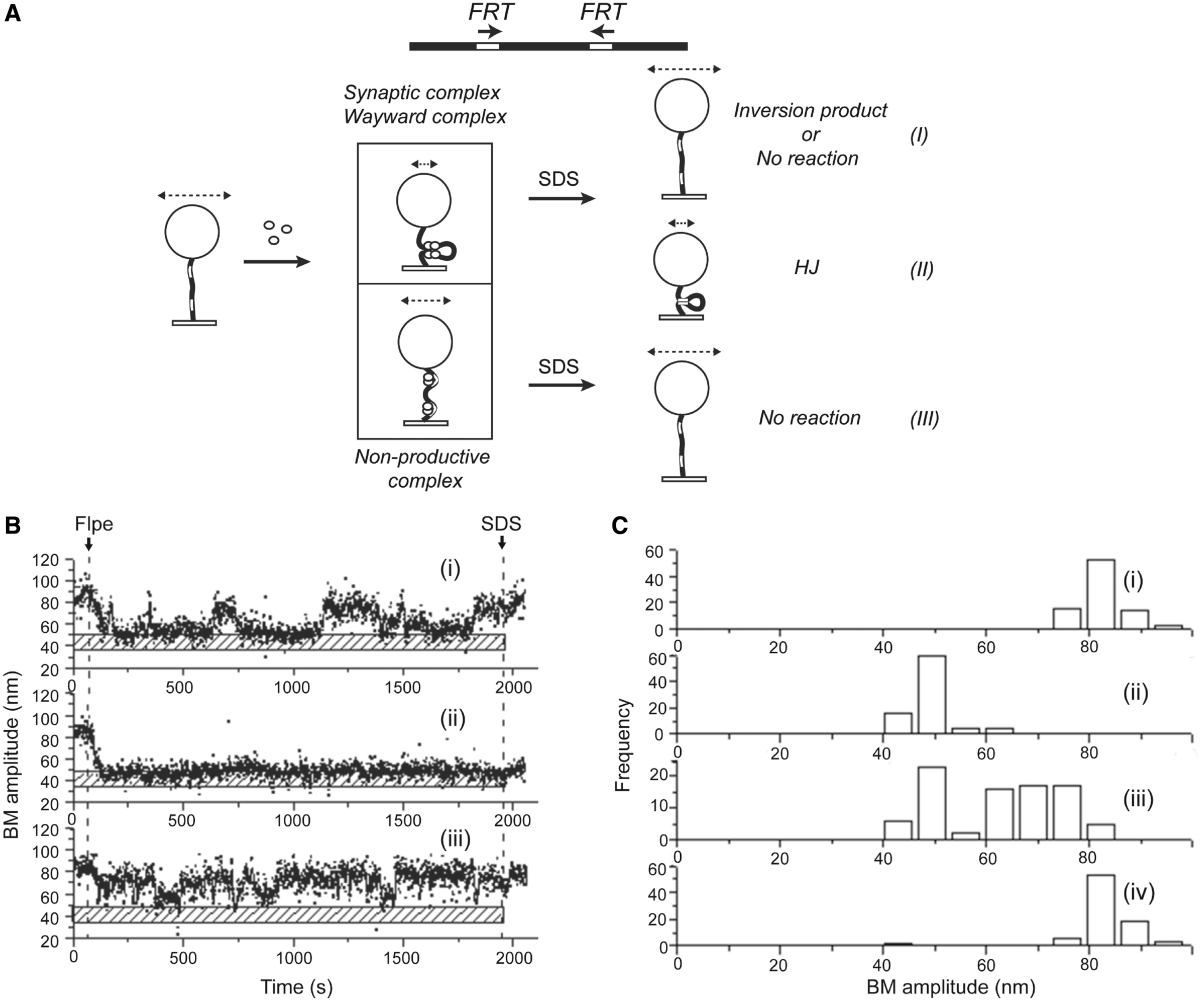
Analysis of Flpe association with *FRT* sites in inverted orientation, synapsis and Holliday junction formation*.* (**A**) Association of Flpe with inverted *FRT* sites in a substrate molecule (BM amplitude = 82.1 ± 8.3 nm) can produce a synaptic complex (BM amplitude = 48.3 ± 6.7 nm) or a non-productive complex (BM amplitude = 59.2 ± 4.8 nm). SDS challenge reinstates an unreacted (wayward) complex or a mature recombinant within the synapse to the high BM amplitude of an Flpe-free parental DNA molecule (I). A non-productive complex is also restored to this BM amplitude (III) on SDS treatment. However, the Holliday junction intermediate retains the same low BM amplitude as the synapsed complex (II). (**B**) The single molecule traces (i), (ii) and (iii) placed alongside the stippled bar indicating the synapsed state (or the Holliday junction) were representative of the outcomes (i), (ii) and (iii), respectively, depicted in A. (**C**) BM amplitude distributions: before Flpe addition (82.1 ± 8.3 nm) (i); in response to Flpe addition (59.2 ± 4.8 nm for non-productive complexes and 48.3 ± 6.7 nm for synaptic complexes) (ii); after 30-min incubation with Flpe (iii); and after SDS challenge at 30 min (iv) (*N* = 86).

According to previous single molecule characterization of λ Int and Cre-mediated recombination reactions, the Holliday junction is a rate-limiting intermediate in the reaction pathway (34,35). The most recent findings from TFM-FRET assays suggest that the Cre reaction pathway is constrained by a slow step following the isomerization of the Holliday junction intermediate (36). Furthermore, in the case of Cre, the relative stability of the Holliday junction, combined with the reversibility of strand cleavage and ligation, affords the opportunity for junction resolution to occur in the parental or the recombinant mode (35). We wished to address the role of the Holliday junction intermediate in determining the kinetics and directionality of its resolution during Flp-mediated recombination. As already noted, for the direct orientation of the recombination sites, reduction in BM amplitude that withstands SDS challenge cannot distinguish Holliday junction formation from the completion of recombination. However, this ambiguity can be resolved when the *FRT* sites are in inverted orientation (Figure 3A). In this case, recombination inverts the DNA orientation between the sites but causes no net change in DNA length. As illustrated in Figure 3A, the DNA tether to the bead is effectively shortened by the Holliday junction but not by the inversion product, once Flpe is dissociated from it.

To estimate the fraction of molecules trapped as Holliday junctions during Flp-mediated recombination, we carried out the TPM assays in 1168 bp DNA molecules containing *FRT* sites in the inverted orientation. The inter-*FRT* spacing was kept the same as in the direct *FRT* sites used in the preceding experiments. Among molecules that associated with Flpe, synapsis of the inverted *FRT*s or formation of non-productive complexes was signaled by characteristic reductions from the initial BM amplitude (Figure 3A). Restoration of the low BM amplitude of the synapse to the high pre-Flpe addition value after SDS challenge denoted events that either completed recombination (indicating synaptic complexes) or failed at recombination (indicating wayward complexes) (Figure 3A-I). The length of a parental molecule would be the same as that of a recombinant molecule resulting from DNA inversion. Retention of the low BM amplitude in the face of SDS challenge attested to the reaction being trapped at the Holliday junction stage (Figure 3A-II). Molecules that formed non-productive complexes were characterized by a persistent higher BM amplitude than the molecules in which the *FRT* sites were synapsed. They switched to the BM amplitude of parental protein-free DNA molecules by spontaneous (before SDS challenge) or forced (after SDS challenge) dissociation of Flpe (Figure 3A-III). Time traces of molecules representing these alternative possibilities are shown in Figure 3B-i–iii.

Of the 117 molecules with a starting BM value of 82.1 ± 8.3 nm (Figure 3C-i) analyzed for their response to Flpe, 86 exhibited a change in the BM amplitude, indicating the interaction between Flpe and the *FRT* sites. Of these 86 molecules, three were trapped as non-productive complexes (that failed to synapse) as indicated by a BM amplitude of 59.2 ± 4.8 nm (Figure 3C-ii). The others formed synaptic complexes typified by an average BM value of 48.3 ± 6.7 nm (Figure 3C-ii). Approximately half the molecules in this population retained this low BM amplitude at 30 min, whereas the others shifted to a higher BM state (Figure 3C-iii). This change was consistent with a dissociation of the synapse before or following the inversion reaction by Flp. On SDS challenge, the starting BM value of 82.1 ± 8.3 nm was restored in most molecules (Figure 3C-iv), indicating that few molecules were present as Holliday junctions at this time point. The outcomes from a shorter 10-min reaction paralleled those from the 30-min reaction and are shown in Supplementary Figure S2A.

Appropriate dwell times fitted to a single exponential model provided the following kinetic features of the reaction. The association constants for the non-productive and pre-synaptic complexes, based on dwell times in the interval between Flpe addition and reduction in BM amplitudes, were (9.8 ± 1.5) × 10^4 ^M^−^^1 ^s^−^^1^ and (7.0 ± 0.3) × 10^4 ^M^−^^1 ^s^−^^1^, respectively (Supplementary Figure S2B-i and ii). The rate constant for the formation of the synaptic complexes, based on the dwell times in the pre-synaptic state, was (2.5 ± 0.2) × 10^−^^2 ^s^−^^1^ (Supplementary Figure S2C-i). The rate constant for the decay of the non-productive complexes based on dwell times in this state was (1.9 ± 0.1) × 10^−^^2 ^s^−^^1^ (Supplementary Figure S2C-ii).

The combined TPM data from the direct and inverted *FRT* sites highlight the robustness of recombination and the transience of the Holliday junction intermediate. Once the junction is formed, its isomerization and resolution follow in quick succession. Furthermore, previous observations suggest that two Flp monomers bound to an *FRT* site can induce strand cleavage even before synapsis (46). Taken together, these results suggest that the formation of the Holliday junction intermediate is a rate-limited event before the downstream steps of recombination. However, according to a previous kinetic analysis of Flp recombination, the rate of the overall reaction is comparable with that of Holliday junction resolution (47). If so, the rate-limiting step in recombination cannot be before junction formation. This apparent discrepancy may arise from differences in the two methodologies. The TPM assays do not disrupt the continuity of the reaction steps; by contrast, the estimated rate of junction resolution is from the purified Holliday intermediate as substrate.

The inclusion of both the direct and inverted *FRT* sites under a common interpretation is justified, as the local organization and function of the recombination synapse would be independent of the global orientation of sites. Indeed, recombination between a pair of symmetrized *FRT* sites results in either DNA inversion or excision with roughly equal probability (48,49). With the ∼600 bp separation between the *FRT* sites in the TPM assays, little difference between direct and inverted sites is expected in their freedom to synapse. This is so, as borne out by recombination yields in ensemble solution assays using the DNA substrates used for the TPM experiments (Supplementary Figure S3).

### Kinetic analysis reveals the execution of a Flp recombination reaction without the reversibility of the intermediate chemical steps

An important feature of the attL × attR reaction by λ Int revealed by TPM analysis is the irreversibility of almost every initiated recombination event until its completion, despite the chemical reversibility of its individual steps expected from purely energetic considerations (34). This uni-directionality from synapsis to excision, provided by the DNA–protein and protein–protein interactions as well as the conformational dynamics within the synaptic complex, is consistent with the physiological purpose of recombination, namely, a switch in the developmental program of the phage. By contrast, a similar analysis of Cre recombination demonstrated the reaction to be reversible in its chemical steps (35). The high efficiency of recombination by Flpe, together with the scant occurrence of the Holliday junction intermediate, observed in the present study (Figures 2D and 3C) suggests that reversibility at intermediate stages of the reaction is unlikely.

The distinct kinetic and thermodynamic behaviors of recombination systems, which differ in being reversible or irreversible in individual chemical steps, can be recognized by the BM fluctuations of DNA molecules carrying direct versus inverted recombination sites (35). For the direct sites, the jump from the low (synapsed) to the high (non-productive) BM amplitude state denotes the decomposition of wayward complexes (unsuccessful in recombination). For the inverted sites, a similar jump could mean either the decomposition of wayward complexes or a successful recombination event, as the parental and recombinant molecules do not differ in length. This analytical distinction holds only if the intermediate chemical steps from synapsis to product formation occur uni-directionally. Reversibility would result in the interconversion among the wayward complexes, Holliday junctions and recombination products within the synapsed state. Thus, the nature of the molecular species responsible for the low–to-high BM transition would become ambiguous.

**Figure 4.**
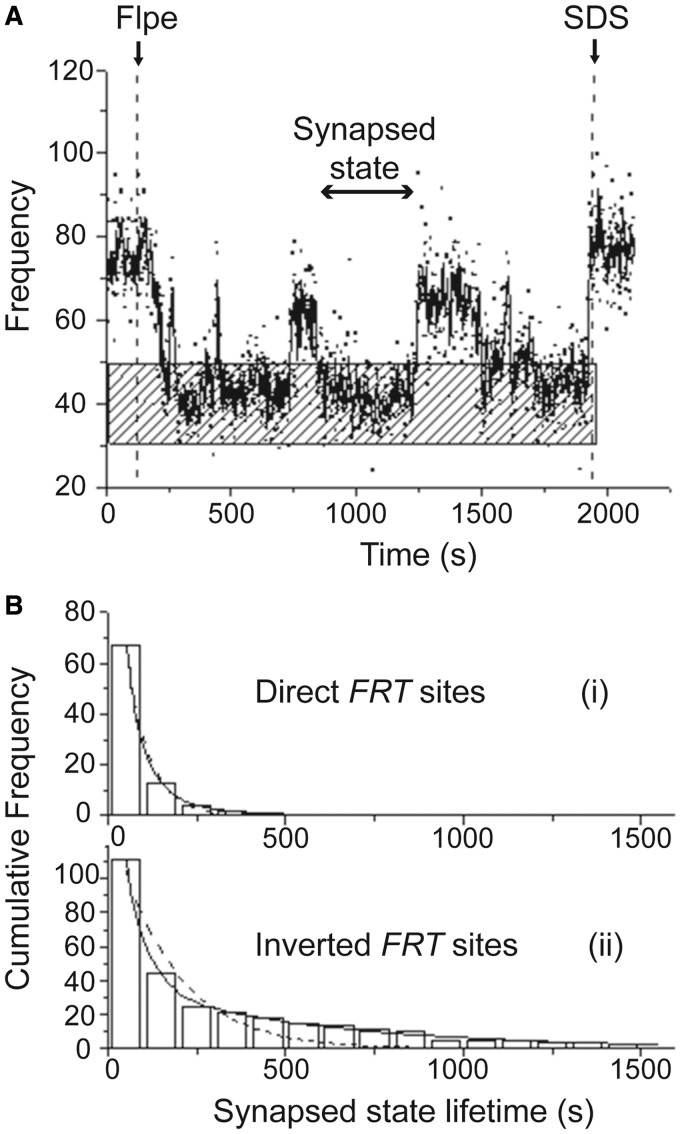
Kinetic analysis of the dwell times in the synapsed state of *FRT* sites in direct and inverted orientations. (**A**) The BM time-trace for a 1168 bp DNA molecule containing direct *FRT* sites in the 30-min interval between Flpe addition and SDS challenge is shown, with the stippled horizontal bar indicating the BM amplitude expected for the synapsed state. The dwell time in a synapsed state, in between two pre-synaptic states, is marked by the line with arrow heads at both ends. (**B**) (i) The histograms were constructed from the pooled distribution of dwell times in the synapsed state revealed by traces similar to that shown in (A). They complied with a single exponential decay model, which gave a rate constant k_1_ = (1.6 ± 0.1) × 10^−2 ^s^−1^ (*R*^2 ^= 1.00; *N* = 67) for the dissociation of synapsed wayward complexes. (ii) The dwell time histograms for inverted *FRT* sites in the synapsed state closely followed a bi-exponential decay model (solid line), and not a single exponential model (dashed line). The rate constants for the bi-exponential decay, k_1_ = (1.7 ± 0.1) × 10^−2 ^s^−1^, A_1_ = 0.85 and k_2_ = (1.7 ± 0.1) × 10^−3 ^s^−1^, A_2_ = 0.15 (*R*^2 ^= 1.00; *N* = 111), pertained to the dissociation of wayward complexes from the synapsed state and the progression of the synapse through the chemical steps of recombination, respectively. The single exponential model gave a rate constant of k_1_ = (6.0 ± 0.8) × 10^−3 ^s^−1^ (R^2 ^= 0.91; *N* = 111).

For *FRT* sites in direct orientation, 19 of 75 DNA molecules displayed BM amplitudes that fluctuated between the synapsed (low BM amplitude) and pre-synaptic (high BM amplitude) states before staying put in the low amplitude state (Figure 4A). SDS challenge showed that most of these molecules had reformed a functional synapse and had undegone Holliday junction formation or excision recombination. Very few, such as the example shown in Figure 4A, remained as parental (or as long-lived wayward complexes). Provided there is no reversal of the steps of recombination, the dwell times in the synapsed state, preceding a switch to the high-amplitude state, should follow a single exponential model, as they represent the stability of only the wayward complexes. The data conformed to this expectation, yielding a dissociation rate constant of (1.6 ± 0.1) × 10^−^^2 ^s^−^^1^ for such complexes (Figure 4B-i). Analogous fluctuations between low and high BM amplitudes mapped for inverted *FRT* sites (Figure 4B-ii) would warrant a bi-exponential model, as they would be correlated not only to the stability of wayward complexes but also to the formation of the inversion product. The experimental results were in agreement with such a model. The rate constants thus derived, (1.7 ± 0.1) × 10^−^^2 ^s^−^^1^ and (1.7 ± 0.1) × 10^−^^3 ^s^−^^1^, represented the dissociation of wayward complexes and recombination activity, respectively.

These kinetic analyses, affirming the fitness of the single and double exponential models in portraying the dwell times of synapsed direct and inverted *FRT* sites, respectively, suggest that the strand cleavage/ligation steps are essentially irreversible during the course of an Flp recombination event. Flp is akin to λ Int (34) but differs from Cre in this respect (35). In the case of Cre, the dwell times of synapsed direct target (*Lox*P) sites fit a double exponential model, suggesting contributions from the decomposition of wayward complexes as well as the reversal of Holliday junction formation.

### Role of strand cleavage in stabilizing the synaptic complex during Flp recombination

It is not clear whether the chemical steps of strand cleavage and/or exchange play a potential role in stabilizing the synaptic complex during tyrosine recombination. Results from the λ Int system suggests that strand cleavage is required for the stability of the synapse (34). However, in the Cre system, the synaptic complex appears to be stable even in the absence of strand cleavage (35). To address how strand cleavage affects synapsis in the Flp system, we used Flpe(Y343F) lacking the cleavage nucleophile Tyr-343. Except for its cleavage incapacity, Flp(Y343) is functionally equivalent to Flp and is fully competent in promoting the joining of a pre-cleaved strand (19,50). Synaptic complexes and wayward complexes cannot be distinguished in the case of Flpe(Y343F) because of its catalytic inactivity.

**Figure 5.**
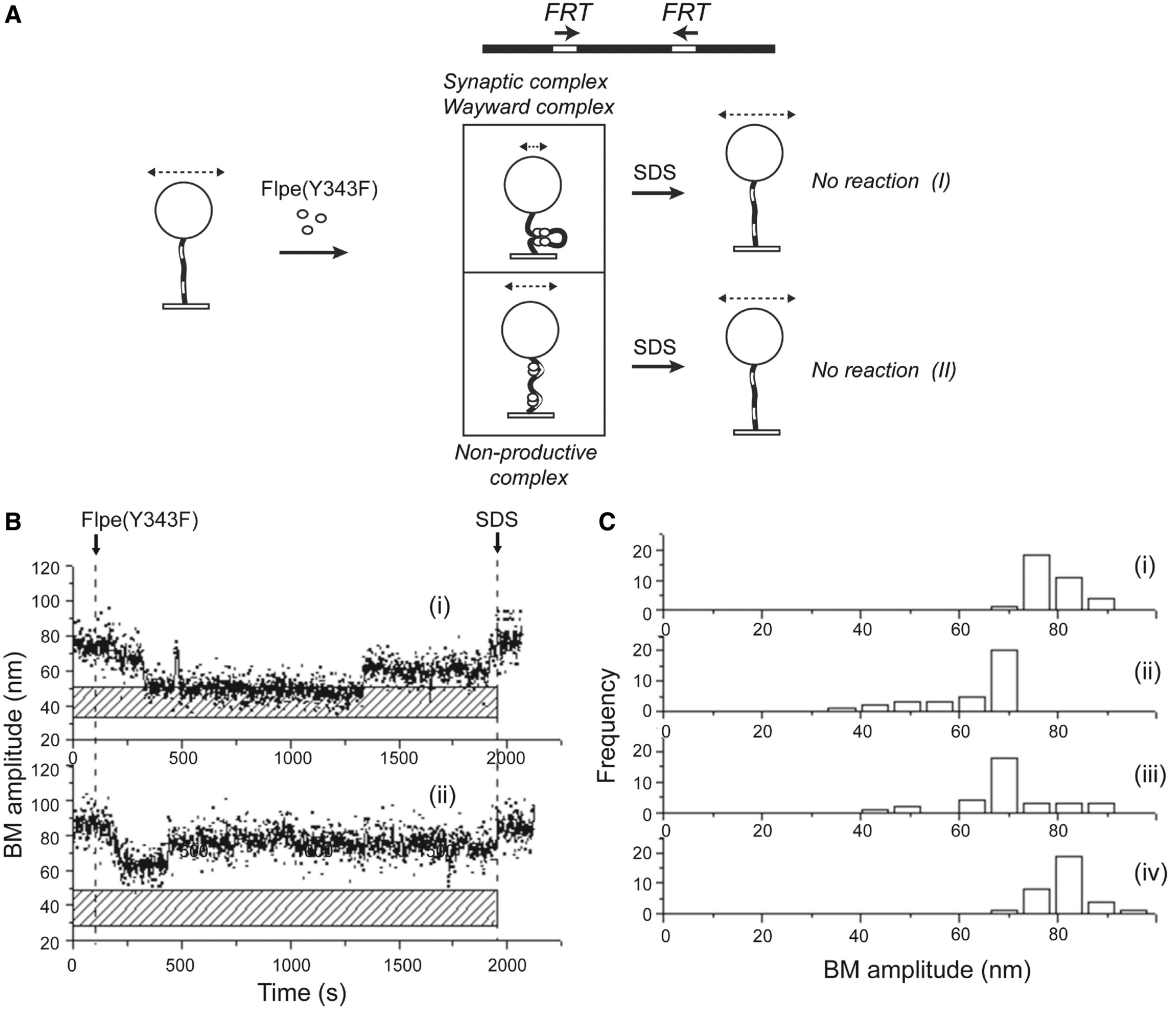
BM amplitude changes induced in 1168 bp DNA molecules containing direct *FRT* sites on association with Flpe(Y343F). (**A**) When DNA molecules (BM amplitude = 78.9 ± 7.9 nm) are occupied by Flpe(Y343F) at the *FRT* sites, they may give rise to synapsed (but wayward) complexes (BM amplitude = 51.0 ± 19.5 nm) or to non-productive complexes (BM amplitude = 66.7 ± 3.6 nm). Consistent with the catalytic inactivity of Flpe(Y343F), SDS challenge reverts both complexes to the parental DNA state (I and II). (**B**) Time traces show a molecule that formed a long-lived, but catalytically inactive (wayward), synapse (i) and one that remained as a non-productive complex for nearly the whole duration of the assay (ii). (**C**) BM amplitude distributions before the addition of Flpe(Y343F) (78.9 ± 7.9 nm) (i); in response to Flpe(Y343F) (66.7 ± 3.6 nm and 51.0 ± 19.5 nm for the non-productive and the synapsed states, respectively) (ii); after a 30-min incubation period (iii); and following SDS challenge at 30 min (iv) (*N* = 34).

The association of Flpe(Y343F) with 1168 bp DNA molecules containing direct *FRT* sites resulted in synapsed complexes or non-productive complexes (Figure 5A) as revealed by time traces of individual molecules (Figure 5B-i and ii). Of the population of DNA molecules analyzed (Figure 5C-i), ∼48.7% was bound by Flpe(Y343F) (Figure 5C-ii). The estimated BM amplitudes suggested that roughly 26.3% of the Flpe(Y343F) bound molecules were in the synapsed state, the rest being non-productive complexes. The low population of synapsed *FRT*s was also evident at 30 min after Flpe(Y343F) addition (Figure 5C-iii). Consistent with the lack of recombination, all molecules showed high BM amplitude following SDS challenge (Figure 5C-iv).

**Figure 6.**
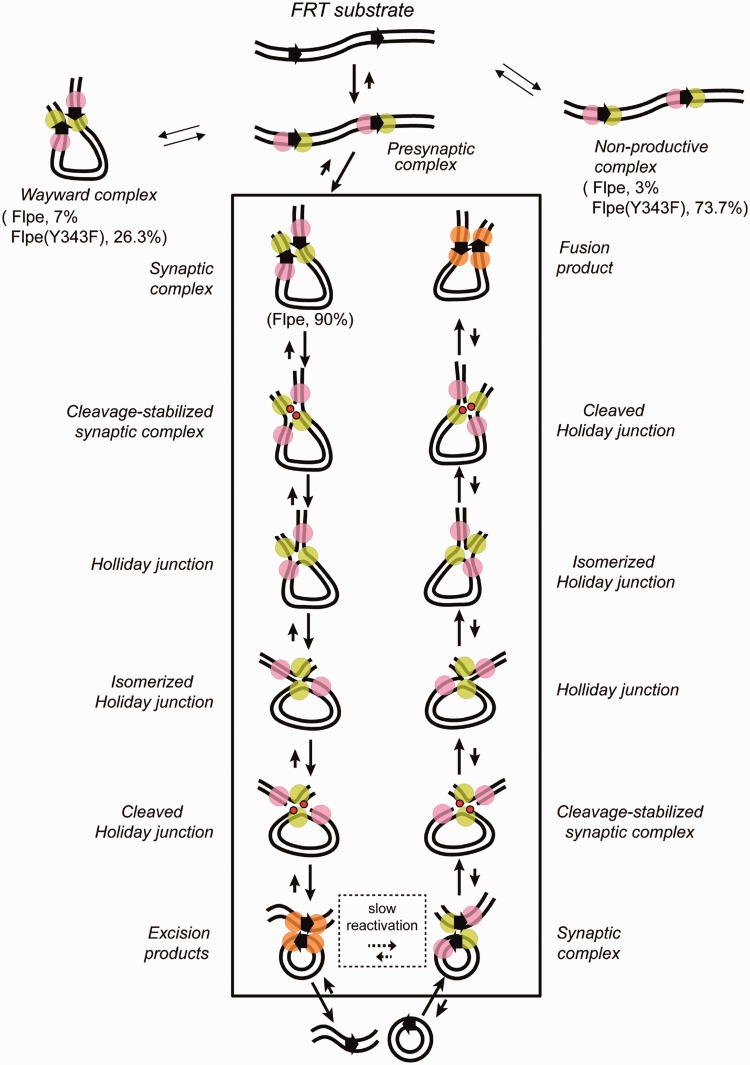
Functional and non-functional Flp-*FRT* associations and the recombination mechanism surmised from the TPM analysis*.* The reaction scheme diagrammed is based on the analyses of Flpe and Flpe(Y343F). Flpe and native Flp almost certainly follow the same chemical mechanism. The pre-chemical steps of recombination shared by Flp and Flp(Y343F) are separated from the conformational and chemical steps of the reaction (exclusive to Flp) within a rectangular boundary. The non-productive and pre-synaptic complexes cannot be distinguished by their BM amplitudes; however, the former do not successfully synapse. The wayward complexes, which share the BM characteristics of the synapsed complexes, fail to complete strand exchange. The Flp reaction is channeled predominantly into the functional recombination pathway. The fraction of Flp-*FRT* associations that are abortive, that is, the sum of non-productive and wayward complexes, is small. This is not the case for Flp(Y343F)-*FRT* associations, as would be consistent with strand cleavage-dependent stabilization of the synapse. The scheme represents the excision reaction with the *FRT* sites shown as direct arrows in the substrate DNA molecule. The Flp monomers bound to them are drawn as paired circles. The scissile phosphates, adjacent to the Flp monomers in green, linked to Tyr-343 during the first and second strand cleavage-exchange steps of recombination are indicated by the red dots. The near irreversibility of the reaction path is thought to result from the conformational modulation of the recombination complex associated with each chemical step. As a result, the synapse that holds together the recombination products may be chemically incompetent (indicated by all four Flp monomers in orange). The reversal of the reaction would require remodeling of the product synapse or establishment of a new synapse.

Single exponential models applied to the dwell time histograms for molecules that responded to Flpe(Y343F) gave association rate constants of (3.1 ± 0.4) × 10^4 ^M^−^^1 ^s^−^^1^ and (1.5 ± 0.2) × 10^4 ^M^−^^1 ^s ^−^^1^ for the formation of non-productive and pre-synaptic complexes, respectively, (Supplementary Figure S4A-i and ii), which were 4–6-fold lower than the corresponding rate constants observed for Flpe (Supplementary Figure S1B-i and ii). The dissociation rate constant for the non-productive complexes formed by Flpe(Y343F) was (2.8 ± 0.3) × 10^−^^2 ^s^−^^1^ (Supplementary Figure S4B-i). Flpe(Y343F) synaptic complexes, chemically inactive and hence wayward, dissociated with a rate constant of (1.3 ± 0.1) × 10^−^^2 ^s^−^^1^ (Supplementary Figure S4B-ii), similar to that estimated for the decomposition of Flpe wayward complexes for both direct and inverted *FRT* systems (Figure 4B-i and ii). The low frequency of synapsed molecules formed by Flpe(Y343F), in conjunction with their propensity to disassemble as frequently as wayward complexes formed by Flpe suggests that strand cleavage contributes to the stability of the Flp recombination synapse. The alternative pathways for the association of Flpe(Y343F) with direct *FRT* sites and the relevant kinetic constants are arranged in Figure 6 and Table 4, respectively.

**Table 4.** The kinetic parameters for the formation and decay of the pre-chemical Flp-DNA complexes and the conversion of synaptic complexes into recombinant products

Wild type or mutant recombinase	Non-productive complex		Wayward complex		Pre-synaptic complex	Synaptic complex	
	k_f_ 10^4 ^M^−1 ^s^−1^	k_d_ 10^−2 ^s^−1^	k_f_ 10^−2 ^s^−1^	k_d_ 10^−2 ^s^−1^	k_f_ 10^4 ^M^−1 ^s^−1^	k_f_ 10^−2 ^s^−1^	k_r_ 10^−3 ^s^−1^
Flpe	18 ± 0.1	4 ± 0.3	1.7 ± 0.2	1.6 ± 0.1	6.0 ± 0.3	4.9 ± 0.4	1.7 ± 0.1
Flpe (Y343F)	3.1 ± 0.4	2.8 ± 0.3	2.2 ± 0.2	1.3 ± 0.1	1.5 ± 0.2	–	–

The rate constants for the individual steps, derived from the dwell times of DNA molecules bound by Flpe or Flpe(Y343F) in the respective precursor states, are indicated.

The rate constants for the formation and decay of a given complex are denoted as kf and kd, respectively.

The rate constant of recombination kr describes the formation of recombinant product(s) from the synaptic complex.

The values for kf and kd are for the excision reaction from direct *FRT* sites diagrammed in Figure 6.

The value for kr is for the inversion reaction from inverted *FRT* sites, based on a double exponential model for the decay of wayward complexes and formation of the recombinant product from the synapsed state (see text for details).

## DISCUSSION

In this study, we have used TPM analysis to reveal the kinetic and thermodynamic features of the Flpe-mediated site-specific recombination from beginning to end (Figure 6 and Table 3). It is reasonable to suppose that these findings apply to native Flp as well. There are similarities and differences in the pre-chemical and chemical steps of recombination between Flp and the mechanistically closely related YRs λ Int and Cre.

### An overview of Flp recombination: Flp-*FRT* associations relevant to recombination

The association of Flp with *FRT* sites predominantly gives rise to pre-synaptic complexes (∼97%) that then go on to organize the recombination synapse (Figure 6). The formation of non-productive complexes under our reaction conditions is infrequent (∼3%). The chemical steps of recombination occur in most synaptic complexes (∼90%), whereas few remain trapped as chemically incompetent wayward complexes (∼7%). In these respects, Flp is broadly similar to λ Int and Cre, whose reaction pathways have been previously analyzed by single molecule TPM (34,35). However, there are also important differences among these individual systems as elaborated later in the text.

The formation of the Int-IHF-Xis complex with the attL and attR sites, which occurs rapidly (>0.05 s^−^^1^), leads quickly to their synapsis (∼0.02 s^−^^1^), and is followed by the quantitative conversion of synapsed molecules into recombinants (34). The formation of the initial protein–DNA complexes, comprising non-productive and pre-synaptic complexes, is slower for the Cre-*Lox*P and Flp-*FRT* systems (35) (this study). The bi-specificity of λ Int in binding the core- and arm-type sequences, and assistance from IHF and Xis in promoting the appropriate DNA conformation (51,52), may account for the speed with which the pre-chemical steps of Int recombination are completed. The association constant for the transition from the pre-synaptic to the synaptic state is 10-fold lower for Flp compared with Cre, (4.9 ± 0.4) × 10^−^^2 ^s^−^^1^ versus (4.2 ± 0.7) × 10^−^^1 ^s^−^^1^ (35). The *FRT* sites used in our studies contain two inverted Flp-binding elements flanking the strand exchange region and constitute the minimal target site required for efficient recombination *in vitro*. Each of the two native *FRT* sites within the two micron circle genome contains a third adjacent Flp-binding element. Perhaps the additional cooperativity provided by a third Flp monomer associated with an *FRT* site may accelerate the steps of pre-synapsis and synapsis *in vivo*. The comparisons of the rates of recombinase association with target sites and of synapse formation made here among λ Int, Cre and Flp are valid, as they were obtained by using similar TPM analyses. However, these rates could be influenced by the force exerted on the DNA by the tethered particle whose motions are tracked (36,53). The association constants for the non-productive complexes and the pre-synaptic state estimated for Flp are within a factor of two of the corresponding values for Cre. However, there is an 8-fold difference in the rate constants for the dissociation of the non-productive complexes between Flp [(4.0 ± 0.3) × 10^−^^2 ^s^−^^1^] and Cre [(5.2 ± 1.0) × 10^−^^3 ^s^−^^1^]. The faster breakdown of the non-productive complexes will increase the overall efficiency of the Flp recombination system by providing a second opportunity to form fresh *FRT*-Flp complexes that may proceed to synapsis. The value of this dissociation constant for Flp obtained from the present TPM analysis is in good agreement with that derived from a previous kinetic study (21).

The formation of wayward complexes within synapsed *FRT* sites may result from the lack of coordination in the establishment of functional interfaces between adjacent Flp subunits (9,44), leading to steric clashes. Our analyses reveal that the wayward complexes dissociate rapidly [(1.7 ± 0.1) × 10^−^^2 ^s^−^^1^] compared with the rate at which the recombination reaction occurs [(1.7 ± 0.1) × 10^−^^3 ^s^−^^1^]. This kinetic difference will also add to recombination efficiency by permitting the wayward complexes additional chances to re-establish a functional synapse.

The synaptic complexes formed by Flp consummate recombination at a high rate of success, with few molecules being trapped as Holliday junctions. Thus, synapsis represents a highly committed step in the Flp and Int reaction pathways. The situation is somewhat different for Cre. Holliday junctions persist as more long-lived intermediates, affording the opportunity for the reaction to reverse course.

### Stability of the Flp-*FRT* recombination synapse: requirement for strand cleavage

As noted earlier, a previous study suggested that synapsis is not a pre-requisite for initiating strand cleavage by Flp (46). In the absence of a compatible DNA partner, the strand nick may be quickly resealed by the ligation reaction. The conformational dynamics within the recombination synapse encourage strand joining across partners, thus leading the system toward recombination. We find that the formation of stable synaptic complexes by Flp is dependent on its ability to perform strand cleavage. A mutation that eliminates the cleavage nucleophile, Y343F, increases the population of non-productive complexes at the expense of synaptic complexes. The cleavage dependent stabilization of the recombination synapse is also the case for λ Int (34), but not for Cre (35).

A previous topological analysis (54) did not reveal strand cleavage as a pre-requisite for synapsis by Flp. By using DNA relaxation by topoisomerase I or ligation of a DNA nick by T4 ligase as a probe for DNA topology, it was demonstrated that Flp(Y343F) introduces a topological change that is commensurate with the synapsis of *FRT* partners. This apparent discrepancy may be due to experimental differences between the topological assays and the TPM analysis. Under the conditions used in the topological studies, the life-time of the Flp(Y343F) established synapse may be sufficiently long relative to the rate of topo I or ligase action. Alternatively, during the TPM assays, the force exerted on the DNA by the movement of the attached polystyrene bead may destabilize the synapse in the absence of strand cleavage (36,53).

### Irreversibility of the chemical steps responsible for an Flp recombination event

The resolution of TPM is limited in that it neither distinguishes the Holliday junction intermediate from the excision product in the case of direct *FRT* sites nor can it differentiate parental substrate from the inversion product in the case of inverted *FRT* sites. However, the combined outcomes from the two orientations of the *FRT* sites tested in separate experiments offset this limitation. BM amplitudes for the parental molecule and the linear excision product are distinct in the case of direct *FRT* sites. Similarly, the Holliday junction intermediate differs in BM amplitude from the parental molecule or the inversion product in the case of inverted *FRT* sites. An important finding from the dual analyses of oppositely oriented pair of *FRT* sites is the near irreversibility of the strand cleavage and ligation steps while a round of Flp recombination is completed.

Kinetic analysis reveals similar rate constants for the dissociation of synapsed wayward complexes formed by Flp with direct and inverted *FRT* sites, (1.6 ± 0.1) × 10^−^^2 ^s^−^^1^ and (1.7 ± 0.1) × 10^−^^2 ^s^−^^1^, respectively. This is also the case for analogous complexes formed by Flp(Y343F) in the one orientation of *FRT* sites tested, namely, direct, (1.3 ± 0.1) × 10^−^^2 ^s^−^^1^. Compared with the dissociation of the wayward complexes, the completion of recombination is a slow event, occurring with a rate constant of (1.7 ± 0.1) × 10^−^^3 ^s^−^^1^, a value that agrees with previous reports (21,55). Consistent with these kinetic attributes, wayward complexes rarely persist within the Flp synapse. Furthermore, Holliday junctions are seldom encountered for the inverted *FRT* sites and the excision product predominates for the direct *FRT* sites. Thus, the passage from synapsis to product is extremely efficient during Flp recombination, owing to the virtually irreversible nature of the intervening strand cleavage and ligation steps. Although such irreversibility is also characteristic of λ Int recombination (34), Cre reaction exhibits reversibility of the chemical steps (35). The difference between Flp and Cre with respect to reversibility is in accord with the observation that the efficiency of excision reaction approaches 100% for Flp recombinase, but does not exceed 75% for Cre (21).

### Reversibility or irreversibility of recombination: physiological implications

Given the isoenergetic nature of the strand cleavage and ligation reactions, the almost uni-directional execution of the steps of a recombination event is remarkable from a chemical perspective. Reversibility is easier to rationalize in the attL × attR reaction by λ Int than *FRT* × *FRT* reaction by Flp. In addition to the DNA–protein and protein–protein interactions responsible for strand cleavage and exchange at the core sites, the attL × attR reaction is driven by additional interactions derived from the association of Int with arm-type sequences and from IHF and Xis bound to their cognate sites. The products of the reaction, attP and attB, differ from attL and attR in their individual sequences, even though the totality of DNA sequence and the number of phosphodiester bonds are conserved. Flp recombination initiated by two substrate *FRT* sites bound by four Flp monomers results in two product *FRT* sites bound by the same four Flp monomers. As suggested by the schemes in Figure 6 and Supplementary Figure S5, additive conformational modulations associated with each sequential chemical step may render the synapse holding the products chemically incompetent, unless a productive synapse is re-established. The synapse resetting process, which might require partial or complete dissociation of the product synapse, may be too slow to be completed within the time scale of the TPM analysis. A combination of the asymmetric DNA bend within the *FRT* sites, the orientations of the tyrosine nucleophiles donated in *trans* to complete active site assembly and the nature of the interface between adjacent Flp monomers may be responsible for this conformational regulation of recombination. Thus, Flp may be able to emulate the λ Int paradigm for irreversibility through a less elaborate set of DNA–protein and protein–protein interactions.

The TPM assays reveal that, under *in vitro* conditions, individual systems differ considerably with respect to reversibility. It is likely that each has been tailored to modulate the physico-chemical and conformational features of the reaction to fulfill the particular biological outcome that it is designed to accomplish. For example, the attL × attR recombination, which excises the λ genome from the bacterial chromosome, is a genetic switch that turns on the lytic program of phage development. Reversibility of the reaction would be counterproductive for the phage. Cre recombination is thought to promote the equal segregation of the unit copy phage/plasmid P1 in its lysogenic state by resolving genome dimers resulting from homologous recombination (1). In principle, reversibility is undesirable in this case as well. It is possible that the *in vitro* Cre reaction fails to recapitulate its native regulatory features. Cre acting *in vivo* on the phage genome organized as a nucleoprotein complex may preferentially promote dimer resolution.

Reversibility, from the recombinant back to the parental state, would be a desirable attribute for Flp to stop potential runaway amplification of the yeast plasmid (34). Amplification is thought to be triggered by the Flp-mediated inversion of a replication fork during bi-directional plasmid replication (2,4). The resulting anti-termination provides a means for multiple copies of the plasmid to be spun out by rolling circle replication. Individual copies of the plasmid may be resolved from the amplified DNA. A second recombination event, which terminates amplification, likely occurs from a newly assembled synapse after the one responsible for initiating amplification has dissociated or has been reset. Reversibility of the chemical steps during a recombination event can only be antithetical to the desired DNA rearrangement in any biological situation.

Recent evidence suggests that post-translational modification of Flp by the host sumoylation system is important in regulating plasmid amplification (56,57), although the mechanism is not understood. Clearly, the bare bones *in vitro* reaction performed on non-chromatin DNA substrates lacks the intricate controls experienced by the *in vivo* reaction. Nevertheless, the *in vitro* system, in conjunction with active site mutants of Flp, provides a useful template for unveiling the conformational and chemical attributes of recombination step by step.

### Flp, λ Int and Cre: evolutionary considerations

λ Int and Cre are produced by unrelated bacterial viruses and serve different biological functions: choice between lysogeny and lysis in the case of λ and dimer resolution and equal segregation in the case of P1. Flp is distinct from both Int and Cre in being housed in a eukaryote and triggers the switch from normal DNA replication to DNA amplification. Flp also differs from the other two in the *trans* organization of its active site. Aside from the assurance that the chemical step of strand cleavage cannot occur until a functional recombinase-target site complex has been assembled, a second potential advantage of the *trans* active site has been suggested (58). In the event that an Flp monomer covalently linked to the cleaved strand via the tyrosine nucleophile becomes denatured or is partially degraded by proteolysis after encountering the replisome, a second Flp monomer has the opportunity to repair the DNA damage by resealing the nick and ejecting the damaged protein.

Our present analyses suggest that Flp is more related to Cre than λ Int in the pre-chemical steps of recombination, whereas the converse is true in the chemical and conformational attributes of the reaction. The general absence of tyrosine recombinases in eukaryotes outside the budding yeast lineage and the fact that the *FLP* gene is housed by an extra-chromosomal element suggest a potential prokaryotic origin of Flp. The two micron plasmid harbors a partitioning system consisting of two partitioning proteins and a partitioning locus assembled from iterations of a consensus sequence element (59). Interestingly, the partitioning system of phage P1 also comprises two partitioning proteins that act in conjunction with an iteron serving as the partitioning locus (60). Despite the organizational similarities, the two partitioning systems are entirely different mechanistically. Nevertheless, it is tempting to speculate that the ancestor of the budding yeast lineage acquired, via horizontal transmission, a genome containing a tyrosine recombination system and a partitioning system from a prokaryotic source. The Flp recombination and the two micron plasmid partitioning systems of today may thus represent the evolutionary transformation of a foreign DNA element during the course of its adaptation to a new biological environment.

## SUMMARY, CONCLUSIONS AND PERSPECTIVES

First, the principal features of the Flp recombination process revealed by TPM analysis are (i) the commitment to recombination concomitant with the synapsis of the *FRT* sites, (ii) the dependence of synapse stability on strand cleavage by Flp and (iii) the irreversibility of strand cleavage and ligation during the course of the reaction (Figure 6). The formation of non-productive complexes and wayward synaptic complexes, signifying aberrant reaction paths, constitutes a minority of events. Second, consistent with previous kinetic studies utilizing λ Int, Cre and Flp (21,27,34–36), the rapid synapsis of *FRT* sites observed by TPM excludes intrinsic barriers that make synapsis by YRs a slow step, at least *in vitro*. Third, the estimated recombination rate and the observed fraction of Holliday junctions within the recombination synapse suggest that formation of this intermediate imposes a slow step before the reaction proceeds further. Under the conditions of the TPM assays, the conformational change responsible for the isomerization of the junction is not a slow process. Forth, the irreversibility of strand cleavage and strand joining during a round of Flp recombination demonstrated by TPM agrees with an earlier finding that the efficiency of the excision reaction by Flp approaches 100% (21). Fifth, Flp is similar to λ Int and differs from Cre, with respect to cleavage dependent stabilization of the synapse and the irreversibility of the strand cleavage and ligation steps responsible for parental-to-recombinant conversion. This is rather unexpected, given the active site organizations of these recombinases. Although Cre and Int assemble their active sites in *cis*, Flp does so in *trans*. Clearly, active site organization is not the primary determinant of how the synapse is stabilized or whether reaction steps can be reversed. Sixth, *in vivo* regulatory functions and DNA organization and topology within the bacterial nucleoid or the budding yeast nucleus are likely to influence the rates of the physical and chemical steps of recombination as well as their directionality.

## SUPPLEMENTARY DATA

Supplementary Data are available at NAR Online: Supplementary Figures 1–5.

## FUNDING

Awards from the National Science Council of Taiwan and National Yang Ming University (to H.F.F.); National Science Foundation grant [MCB-1049925] and a Robert F. Welch Foundation award [F-1274 to M.J.]. Funding for open access charge: National Science Council of Taiwan and National Yang Ming University.

*Conflict of interest statement*. None declared.

## Supplementary Material

Supplementary Data

## References

[1] Austin S, Ziese M, Sternberg N (1981). A novel role for site-specific recombination in maintenance of bacterial replicons. Cell.

[2] Futcher AB (1986). Copy number amplification of the 2 micron circle plasmid of *Saccharomyces cerevisiae*. J. Theor. Biol..

[3] Rajeev L, Malanowska K, Gardner JF (2009). Challenging a paradigm: the role of DNA homology in tyrosine recombinase reactions. Microbiol. Mol. Biol. Rev..

[4] Volkert FC, Broach JR (1986). Site-specific recombination promotes plasmid amplification in yeast. Cell.

[5] Azaro MA, Landy A, Craig NL, Craigie R, Gellert M, Lambowitz AM (2002). λ integrase and λ Int family. Mobile DNA II.

[6] Barre FX, Sherratt DJ, Craig NL, Craigie R, Gellert M, Lambowitz AM (2002). Xer site-specific recombination: promoting chromosome segregation. Mobile DNA II.

[7] Grindley ND, Whiteson KL, Rice PA (2006). Mechanisms of site-specific recombination. Annu. Rev. Biochem..

[8] Ghosh K, Van Duyne GD (2002). Cre-loxP biochemistry. Methods.

[9] Chen Y, Rice PA (2003). New insight into site-specific recombination from Flp recombinase-DNA structures. Annu. Rev. Biophys. Biomol. Struct..

[10] Buchholz F, Ringrose L, Angrand PO, Rossi F, Stewart AF (1996). Different thermostabilities of FLP and Cre recombinases: implications for applied site-specific recombination. Nucleic Acids Res..

[11] Buchholz F, Angrand PO, Stewart AF (1996). A simple assay to determine the functionality of Cre or FLP recombination targets in genomic manipulation constructs. Nucleic Acids Res..

[12] Kuhn R, Schwenk F, Aguet M, Rajewsky K (1995). Inducible gene targeting in mice. Science.

[13] Le Y, Sauer B (2000). Conditional gene knockout using Cre recombinase. Methods Mol. Biol..

[14] Long DP, Zhao AC, Chen XJ, Zhang Y, Lu WJ, Guo Q, Handler AM, Xiang ZH (2012). FLP recombinase-mediated site-specific recombination in silkworm, Bombyx mori. PLoS One.

[15] Venken KJ, Bellen HJ (2012). Genome-wide manipulations of Drosophila melanogaster with transposons, Flp recombinase, and PhiC31 integrase. Methods Mol. Biol..

[16] Takata Y, Kondo S, Goda N, Kanegae Y, Saito I (2011). Comparison of efficiency between FLPe and Cre for recombinase-mediated cassette exchange in vitro and in adenovirus vector production. Genes Cells.

[17] Christ N, Droge P (2002). Genetic manipulation of mouse embryonic stem cells by mutant lambda integrase. Genesis.

[18] Van Duyne GD, Craig NL, Craigie R, Gellert M, Lambowitz AM (2002). A structural view of tyrosine recombinase site-specific recombination. Mobile DNA II.

[19] Chen JW, Lee J, Jayaram M (1992). DNA cleavage in trans by the active site tyrosine during Flp recombination: switching protein partners before exchanging strands. Cell.

[20] Chen Y, Narendra U, Iype LE, Cox MM, Rice PA (2000). Crystal structure of a Flp recombinase-Holliday junction complex: assembly of an active oligomer by helix swapping. Mol. Cell.

[21] Ringrose L, Lounnas V, Ehrlich L, Buchholz F, Wade R, Stewart AF (1998). Comparative kinetic analysis of FLP and Cre recombinases: mathematical models for DNA binding and recombination. J. Mol. Biol..

[22] Du Q, Livshits A, Kwiatek A, Jayaram M, Vologodskii A (2007). Protein-induced local DNA bends regulate global topology of recombination products. J. Mol. Biol..

[23] Guo F, Gopaul DN, Van Duyne GD (1997). Structure of Cre recombinase complexed with DNA in a site-specific recombination synapse. Nature.

[24] Better M, Lu C, Williams RC, Echols H (1982). Site-specific DNA condensation and pairing mediated by the Int protein of bacteriophage lambda. Proc. Natl Acad. Sci. USA.

[25] Hamilton DL, Abremski K (1984). Site-specific recombination by the bacteriophage P1 lox-Cre system. Cre-mediated synapsis of two lox sites. J. Mol. Biol..

[26] Segall AM, Nash HA (1993). Synaptic intermediates in bacteriophage lambda site-specific recombination: integrase can align pairs of attachment sites. EMBO J..

[27] Ghosh K, Guo F, Van Duyne GD (2007). Synapsis of loxP sites by Cre recombinase. J. Biol. Chem..

[28] Vetcher AA, Lushnikov AY, Navarra-Madsen J, Scharein RG, Lyubchenko YL, Darcy IK, Levene SD (2006). DNA topology and geometry in Flp and Cre recombination. J. Mol. Biol..

[29] Cassell G, Moision R, Rabani E, Segall A (1999). The geometry of a synaptic intermediate in a pathway of bacteriophage lambda site-specific recombination. Nucleic Acids Res..

[30] Guo F, Gopaul DN, Van Duyne GD (1999). Asymmetric DNA bending in the Cre-loxP site-specific recombination synapse. Proc. Natl Acad. Sci. USA.

[31] Ennifar E, Meyer JE, Buchholz F, Stewart AF, Suck D (2003). Crystal structure of a wild-type Cre recombinase-loxP synapse reveals a novel spacer conformation suggesting an alternative mechanism for DNA cleavage activation. Nucleic Acids Res..

[32] Rufer A, Neuenschwander PF, Sauer B (2002). Analysis of Cre-loxP interaction by surface plasmon resonance: influence of spermidine on cooperativity. Anal. Biochem..

[33] Shoura MJ, Vetcher AA, Giovan SM, Bardai F, Bharadwaj A, Kesinger MR, Levene SD (2012). Measurements of DNA-loop formation via Cre-mediated recombination. Nucleic Acids Res..

[34] Mumm JP, Landy A, Gelles J (2006). Viewing single lambda site-specific recombination events from start to finish. EMBO J..

[35] Fan HF (2012). Real-time single-molecule tethered particle motion experiments reveal the kinetics and mechanisms of Cre-mediated site-specific recombination. Nucleic Acids Res..

[36] Pinkney JN, Zawadzki P, Mazuryk J, Arciszewska LK, Sherratt DJ, Kapanidis AN (2012). Capturing reaction paths and intermediates in Cre-loxP recombination using single-molecule fluorescence. Proc. Natl Acad. Sci. USA.

[37] Ma CH, Rowley PA, Macieszak A, Guga P, Jayaram M (2009). Active site electrostatics protect genome integrity by blocking abortive hydrolysis during DNA recombination. EMBO J..

[38] Konieczka JH, Paek A, Jayaram M, Voziyanov Y (2004). Recombination of hybrid target sites by binary combinations of Flp variants: mutations that foster interprotomer collaboration and enlarge substrate tolerance. J. Mol. Biol..

[39] Buchholz F, Angrand PO, Stewart AF (1998). Improved properties of FLP recombinase evolved by cycling mutagenesis. Nat. Biotechnol..

[40] Voziyanov Y, Stewart AF, Jayaram M (2002). A dual reporter screening system identifies the amino acid at position 82 in Flp site-specific recombinase as a determinant for target specificity. Nucleic Acids Res..

[41] Liu P, Jenkins NA, Copeland NG (2003). A highly efficient recombineering-based method for generating conditional knockout mutations. Genome Res..

[42] Fan HF, Li HW (2009). Studying RecBCD helicase translocation along Chi-DNA using tethered particle motion with a stretching force. Biophys. J..

[43] Fan HF, Cox MM, Li HW (2011). Developing single-molecule TPM experiments for direct observation of successful RecA-mediated strand exchange reaction. PLoS One.

[44] Conway AB, Chen Y, Rice PA (2003). Structural plasticity of the Flp-Holliday junction complex. J. Mol. Biol..

[45] Luetke KH, Sadowski PD (1995). The role of DNA bending in Flp-mediated site-specific recombination. J. Mol. Biol..

[46] Voziyanov Y, Lee J, Whang I, Lee J, Jayaram M (1996). Analyses of the first chemical step in Flp site-specific recombination: synapsis may not be a pre-requisite for strand cleavage. J. Mol. Biol..

[47] Meyer-Leon L, Inman RB, Cox MM (1990). Characterization of Holliday structures in FLP protein-promoted site-specific recombination. Mol. Cell. Biol..

[48] Grainge I, Pathania S, Vologodskii A, Harshey RM, Jayaram M (2002). Symmetric DNA sites are functionally asymmetric within Flp and Cre site-specific DNA recombination synapses. J.Mol. Biol..

[49] Senecoff JF, Cox MM (1986). Directionality in FLP protein-promoted site-specific recombination is mediated by DNA-DNA pairing. J. Biol. Chem..

[50] Pan G, Luetke K, Juby CD, Brousseau R, Sadowski P (1993). Ligation of synthetic activated DNA substrates by site-specific recombinases and topoisomerase I. J. Biol. Chem..

[51] Radman-Livaja M, Biswas T, Ellenberger T, Landy A, Aihara H (2006). DNA arms do the legwork to ensure the directionality of lambda site-specific recombination. Curr. Opin. Struct. Biol..

[52] Biswas T, Aihara H, Radman-Livaja M, Filman D, Landy A, Ellenberger T (2005). A structural basis for allosteric control of DNA recombination by lambda integrase. Nature.

[53] Bai H, Kath JE, Zorgiebel FM, Sun M, Ghosh P, Hatfull GF, Grindley ND, Marko JF (2012). Remote control of DNA-acting enzymes by varying the Brownian dynamics of a distant DNA end. Proc. Natl Acad. Sci. USA.

[54] Grainge I, Buck D, Jayaram M (2000). Geometry of site alignment during int family recombination: antiparallel synapsis by the Flp recombinase. J. Mol. Biol..

[55] Gates CA, Cox MM (1988). FLP recombinase is an enzyme. Proc. Natl Acad. Sci. USA.

[56] Xiong L, Chen XL, Silver HR, Ahmed NT, Johnson ES (2009). Deficient SUMO attachment to Flp recombinase leads to homologous recombination-dependent hyperamplification of the yeast 2 micron circle plasmid. Mol. Biol. Cell.

[57] Chen XL, Reindle A, Johnson ES (2005). Misregulation of 2 micron circle copy number in a SUMO pathway mutant. Mol. Cell. Biol..

[58] Rice PA, Craig NL, Craigie R, Gellert M, Lambowitz AM (2002). Theme and variation in tyrosine recombinases: structure of a Flp-DNA complex. Mobile DNA II.

[59] Jayaram M, Yang XM, Mehta S, Voziyanov Y, Velmurugan S, Funnell BE, Phillips G (2004). The 2 micron plasmid of *Saccharomyces cerevisiae*. Plasmid Biology.

[60] Funnell BE, Slavcev RE, Funnell BE, Phillips G (2004). Partition systems of bacterial plasmids. *Plasmid* Biology.

