# Supplementary material for: Real-time single-molecule tethered particle motion analysis reveals mechanistic similarities and contrasts of Flp site-specific recombinase with Cre and λ Int
Source: Nucleic Acids Res. 2013 May 21;41(14):7031–47. doi: 10.1093/nar/gkt424 (PMC3737535; doi:10.1093/nar/gkt424)
Supplement: Supplementary Data [file supp_41_14_7031__index.html]

Real-time single-molecule tethered particle motion analysis reveals mechanistic similarities and contrasts of Flp site-specific recombinase with Cre and λ Int — Real-time single-molecule tethered particle motion analysis reveals mechanistic similarities and contrasts of Flp site-specific recombinase with Cre and λ Int — Supplementary Data 

# Real-time single-molecule tethered particle motion analysis reveals mechanistic similarities and contrasts of Flp site-specific recombinase with Cre and λ Int

## Supplementary Data

files

**Files in this Data Supplement:**

- Supplementary Data - pdf file
