# Supplementary material for: Real-time single-molecule tethered particle motion analysis reveals mechanistic similarities and contrasts of Flp site-specific recombinase with Cre and λ Int
Source: Nucleic Acids Res. 2013 May 21;41(14):7031–47. doi: 10.1093/nar/gkt424 (PMC3737535; doi:10.1093/nar/gkt424)
Supplement: Supplementary Data [file supp_gkt424_nar-00837-h-2013-File002.pdf]

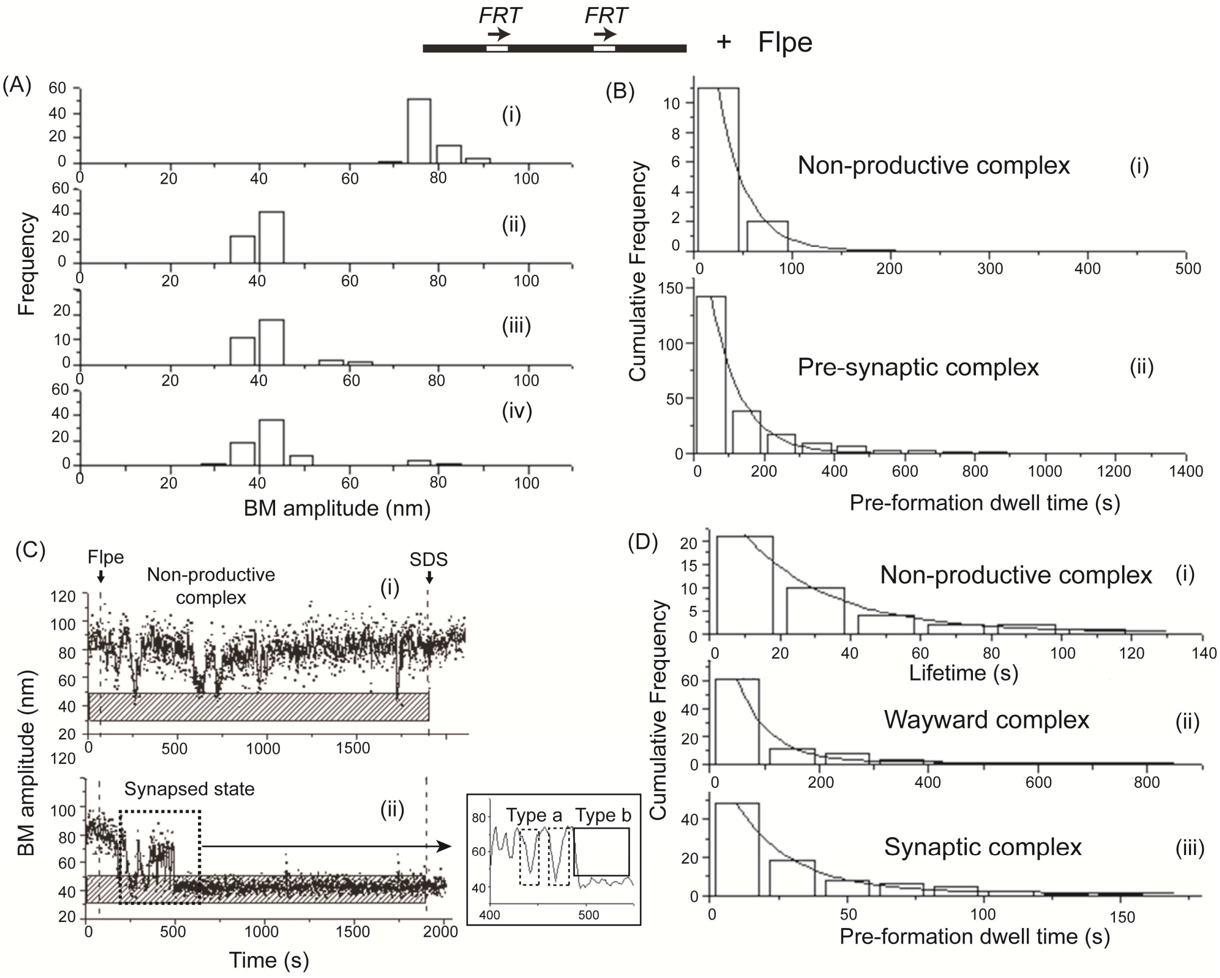

Figure S1. *Flpe association and recombination events in 1168 bp DNA molecules containing direct FRT sites over a 10 min time course.* The analyses were similar to those assembled in Fig. 2, except that the time course was shortened to 10 min. (A) BM amplitudes before Flpe addition (78.0 ± 7.4 nm) (i); in response to Flpe addition (39.7 ± 2.9 nm for the synapsed state) (ii); at 10 min of incubation with Flpe (iii); and after SDS challenge at the end of 10 min incubation (iv) (N = 36). (B) The distribution of the dwell times between Flpe addition and the change in the BM amplitude were fitted to a single exponential algorithm (Origin 8.0) to obtain the association rate constants, $(1.8 \pm 0.1) \times 10^5$ M^{-1} s^{-1} ($R^2 = 1.00$, N = 11) for the non-productive complexes (i) and

$(6.0 \pm 0.3) \times 10^4$ M^{-1} s^{-1} ($R^2 = 0.99$, N = 142) for the pre-synaptic complexes (ii). (C) BM time-traces depicting responses to Flpe addition are shown, with the stippled bar representing the synapsed state. (i) A molecule that was trapped as a non-productive complex; (ii) a molecule that formed a stable synapse (Type b) after two episodes of transient synapse (Type a). (D) The dwell times of molecules in the non-synapsed state or the pre-synaptic state were used in a single exponential model to obtain the following rate constants: $(4.0 \pm 0.3) \times 10^{-2}$ s^{-1} ($R^2 = 0.99$, N = 21) for the decay of the non-productive complexes (i); $(1.7 \pm 0.2) \times 10^{-2}$ s^{-1} ($R^2 = 0.99$, N = 48) for the formation of the transiently synapsed wayward complexes (ii; Type a in C-ii) and (4.9 ± 0.4) $\times 10^{-2}$ s^{-1} ($R^2 = 0.99$, N = 61) for the formation of the stable synaptic complexes (iii; Type b in C-ii). The level of significance for the data in this figure and others to follow was $p < 0.05$.

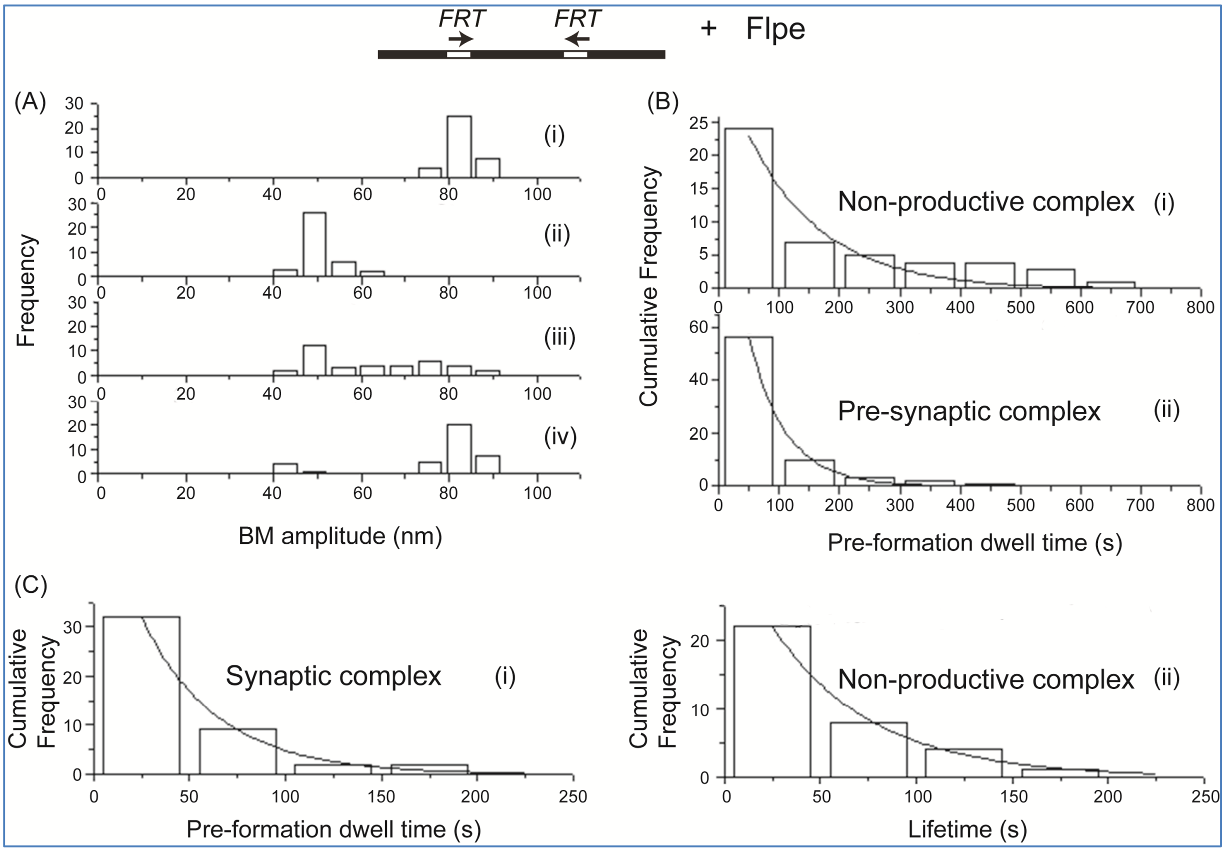

Figure S2. *Flpe association and recombination events in 1168 bp DNA molecules containing inverted FRT sites over a 10 min time course.* (A) The BM amplitude distributions before Flpe addition (83.0 ± 7.6 nm) (i), in response to Flpe addition (58.7 ± 4.8 nm for the non-productive complexes and 49.2 ± 6.2 nm for the synaptic complexes) (ii), after 10 min incubation with Flpe (iii) and after SDS challenge at 10 min (iv) are presented (N = 55). (B) The dwell times between Flpe addition and changes in the BM amplitude yielded association rate constants of (9.8 ± 1.5) × 10^4 M^{-1} s^{-1} (R^2 = 1.00, N = 24) for the non-productive complexes (i) and (7.0 ± 0.3) × 10^4 M^{-1} s^{-1} (R^2 = 1.00, N = 56) for the pre-synaptic complexes (ii). (C) The dwell times in the presynaptic state (i) gave an association rate constant of (2.5 ± 0.2) × 10^{-2} s^{-1} (R^2 = 1.00, N = 32) for assembly of the synaptic complex. The dwell times in the nonproductive complex gave the rate constant for its decay as (1.9 ± 0.1) × 10^{-3} s^{-1} (R^2 = 1.00, N = 25).

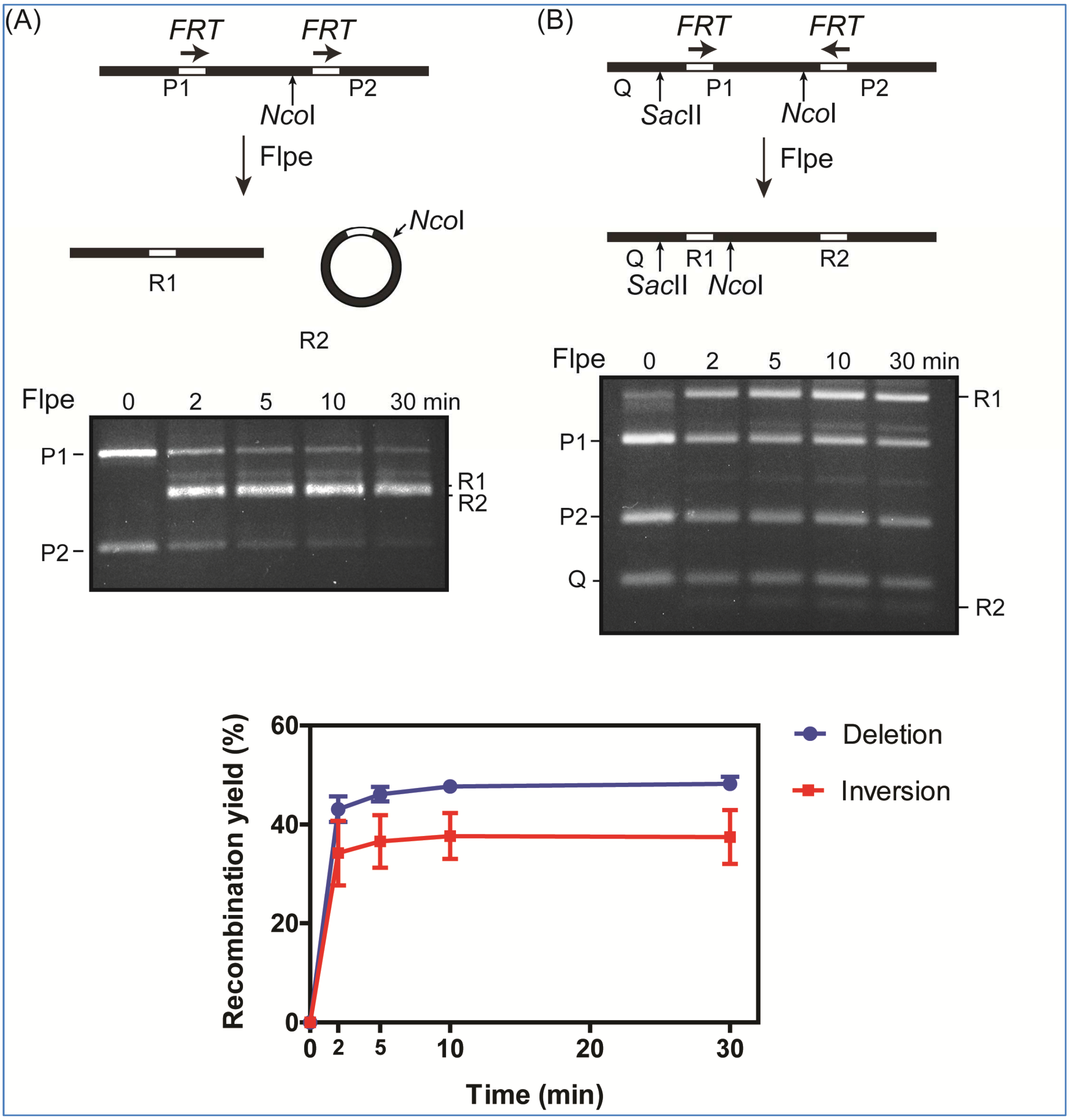

Figure S3. *Recombination yields for excision and inversion reactions by Flpe*. The linear recombination substrates containing a pair of *FRT* sites in the direct (**A**) or inverted (**B**)

orientation were the same as those on which the TPM assays were performed (see Figures 2 and 3; also Figures S1 and S2 above). The outcomes of the Flpe mediated excision and inversion are schematically illustrated at the top. The recombination reactions were carried out for the indicated times at room temperature (22°C) using the same Flpe preparation and buffer conditions employed in the TPM assays. The number of Flp monomers per Flp binding element was kept at approximately 8-10 in order to saturate Flp-*FRT* association. After processing the reactions, the DNA from the excision reaction was digested with NcoI to linearize the circular product, and that from the inversion reaction with NcoI plus SacII to distinguish the inversion product from the substrate. The digested DNA was subjected to electrophoresis in 1.5% agarose, and visualized by ethidium bromide staining. The intensities of the bands were quantitated using Quantity One-4.6.5 image analysis software (Bio-Rad). The yield of excision in each lane was estimated as the sum of the intensities of the recombinant bands (R1 + R2) divided by the sum of the intensities of the parental (P) and recombinant bands (P + R1 + R2). Similarly, the yield of inversion was estimated as (R1 + R2) divided by (P1 + P2 + R1 + R2), where P1 and P2 are the parental bands. The third band common to both the parent and the recombinant (Q) was omitted from the calculation. In estimating R1 intensities, the intensity of the faint band in the control (leftmost) lane that partially overlaps with it was subtracted. The stray band was caused by a minor byproduct from the PCR reaction by which the inversion substrate was prepared. The percent recombination as a function of time, averaged from four separate assays, was graphed for the excision and inversion reactions. The error bars represent standard deviations of the mean.

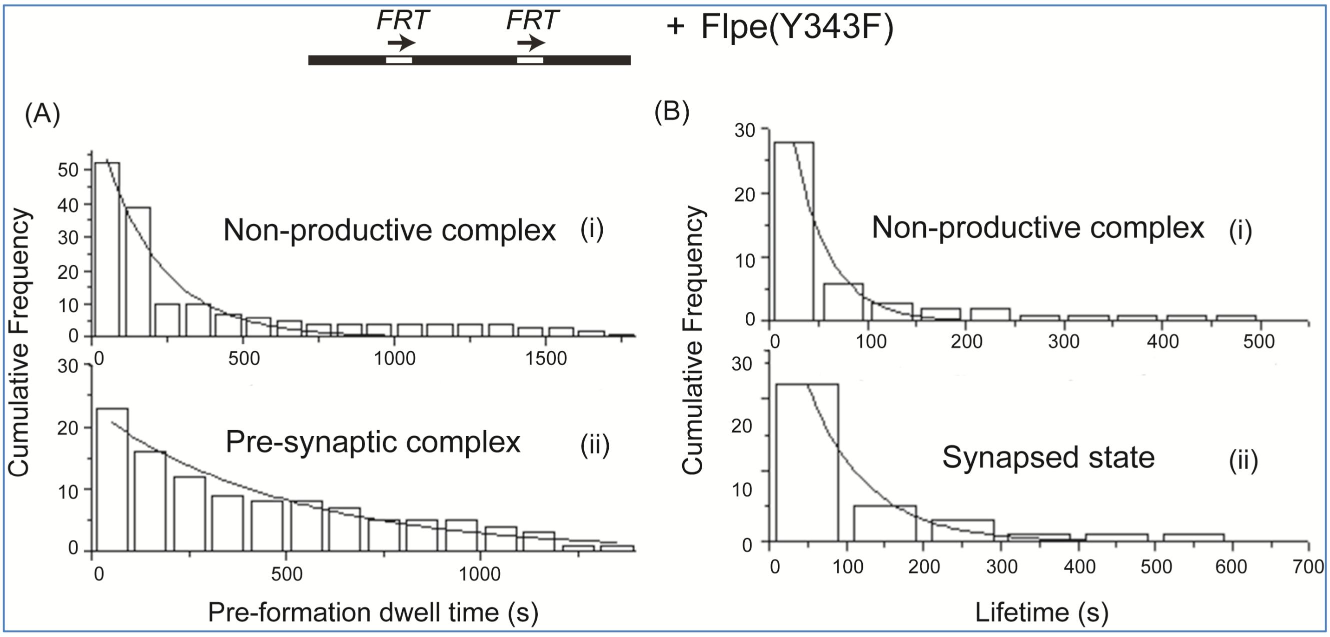

Figure S4. *Flpe(Y343F) complex formation in 1168 bp DNA molecules containing direct FRT sites.* (A) The dwell time histograms in the interval between Flpe(Y343F) addition and BM amplitude changes corresponded to an association rate constant of (3.1 ± 0.4) × 10^4 M^{-1} s^{-1} (R^2 = 0.99, N = 52) for the non-productive complexes and (1.5 ± 0.2) × 10^4 M^{-1} s^{-1} (R^2 = 0.99, N = 23) for the pre-synaptic complexes. (B) (i) The dwell times of the nonproductive complexes gave their single exponential decay constant as (2.8 ± 0.3) × 10^{-2} s^{-1} (R^2 = 1.00, N = 28). (ii) The dwell times of molecules in the wayward synaptic state yielded, according to single exponential decay, their dissociation rate constant as (1.3 ± 0.1) × 10^{-2} s^{-1} (R^2 = 1.00, N = 22).

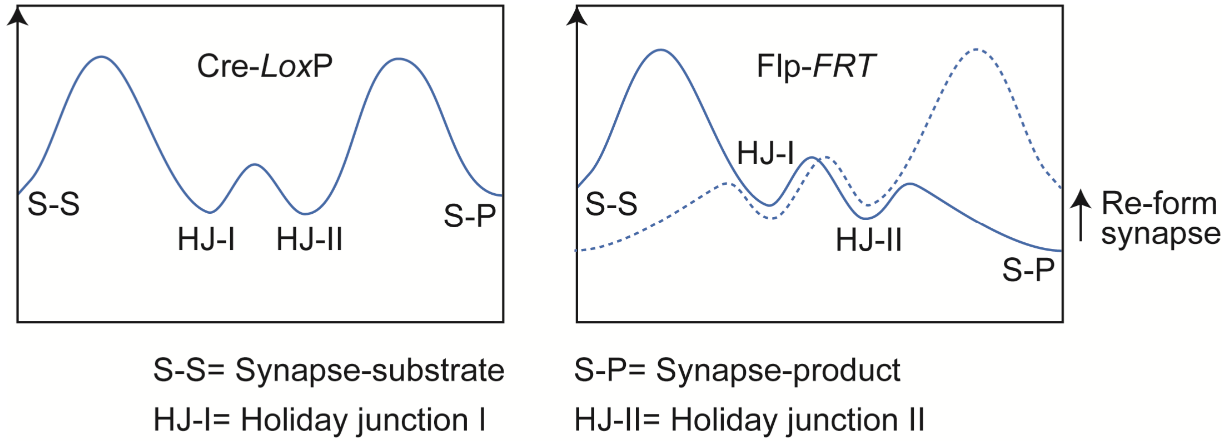

Figure S5. Energetics of the Cre-*Lox*P and Flp-*FRT* recombination pathways. Reaction coordinate versus energy diagrams representing Cre and Flp mediated recombination reactions are purely schematic, and are intended only to denote reversibility in the former case, and the near lack of reversibility in the latter. The reaction proceeds via the synapsis of recombinase bound substrate sites, formation of Holliday junction (HJ-I), its isomerization to HJ-II and the resolution of HJ-II to products. The products are also held together in a synapsis-like state. In the Cre-*Lox*P system, with a symmetric energy profile, the Holliday junction introduces an energetic trap. In the Flp-*FRT* system, the reaction follows an overall downhill path, facilitated, in part, by a cleavage dependent stabilization of the substrate synapse. The product *FRT* sites formed by resolution of HJ-II are held within a non-functional synapse. This synapse has to be remodeled to the functional state or re-formed before the reaction can proceed along the energy path depicted by the dashed line.
